# Wide range zero-thermal-quenching ultralong phosphorescence from zero-dimensional metal halide hybrids

**DOI:** 10.1038/s41467-020-18482-w

**Published:** 2020-09-16

**Authors:** Shuya Liu, Xiaoyu Fang, Bo Lu, Dongpeng Yan

**Affiliations:** 1 Beijing Key Laboratory of Energy Conversion and Storage Materials, College of Chemistry, Beijing Normal University, 100875 Beijing, P. R. China; 2 Key Laboratory of Theoretical and Computational Photochemistry, Ministry of Education, College of Chemistry, Beijing Normal University, 100875 Beijing, P. R. China

**Keywords:** Optical materials, Photochemistry, Crystal engineering, Applied optics

## Abstract

Materials with ultralong phosphorescence have wide-ranging application prospects in biological imaging, light-emitting devices, and anti-counterfeiting. Usually, molecular phosphorescence is significantly quenched with increasing temperature, rendering it difficult to achieve high-efficiency and ultralong room temperature phosphorescence. Herein, we spearhead this challenging effort to design thermal-quenching resistant phosphorescent materials based on an effective intermediate energy buffer and energy transfer route. Co-crystallized assembly of zero-dimensional metal halide organic-inorganic hybrids enables ultralong room temperature phosphorescence of (Ph_4_P)_2_Cd_2_Br_6_ that maintains luminescent stability across a wide temperature range from 100 to 320 K (ΔT = 220 °C) with the room temperature phosphorescence quantum yield of 62.79% and lifetime of 37.85 ms, which exceeds those of other state-of-the-art systems. Therefore, this work not only describes a design for thermal-quenching-resistant luminescent materials with high efficiency, but also demonstrates an effective way to obtain intelligent systems with long-lasting room temperature phosphorescence for optical storage and logic compilation applications.

## Abstract (web summary)

Molecular luminescence is generally significantly quenched at high temperature. Here the authors report a zero-dimensional metal halide hybrid that exhibits zero-thermal-quenching ultralong phosphorescence over a wide temperature range of 220 K, and demonstrate use for Morse code encryption and logic gates.

## Introduction

Molecular luminescent materials have attracted increasingly widespread attention due to their extensive applications in illumination resources, light-emitting diodes (LEDs), and biological imaging among many others^1–20^. It is well known that the luminescence of molecular systems is usually sensitive to temperature, such that increasing temperature tends to provoke with intensification of molecular rotation and lattice vibration in chromophores, thereby facilitating the universal thermal-quenching (TQ) effect^21,22^. For example, such thermal effects are obstacles to high luminescent efficiency and therefore restrict the practical suitability of phosphors for commercial applications involving LEDs at high temperature. Quite recently, it was demonstrated that the introduction of intermediate energy levels or defect states for energy transfer in pure inorganic phosphors can diminish the TQ effect to some extent^23–27^; however, examples of materials with zero-TQ emission are still rather rare, particularly for the case of molecular systems (Supplementary Table 1)^28–30^. Therefore, obtaining TQ-resistant materials remains an open challenge.

The origin of phosphorescence usually involves a photoluminescence (PL) transition between the excited triplet and singlet states. The forbidden transition renders molecular phosphorescence generally observable at very low temperatures, but difficult to achieve at ambient conditions^31^. Until recently, molecules with long-lived room-temperature phosphorescence (RTP) garnered much interest due to their potential applications in displays, anti-counterfeiting, and information storage^22,32–40^. To date, despite continuous reports of RTP systems with ultralong lifetimes ranging from the microsecond (ms) to second (s) timescale, such materials with high RTP efficiency are still quite limited, since pronounced quenching of the triplet excitons occurs via nonradiative transitions at elevated temperature^21,22^. Considering the long-standing scientific and technological demands for simultaneous thermally resistant luminescence and ultralong RTP with high efficiency, the facile design of materials with zero TQ phosphorescence could greatly enrich the RTP family, and accordingly enhance the achievable quantum yield. However, such systems are still merely speculated.

Low-dimensional organic–inorganic hybrid metal halides have exhibited novel optoelectronic properties in applications, such as perovskite solar cells and photoemission devices^41,42^. For example, in zero-dimensional (0D) metal-halide systems, isolation of the inorganic polyhedral units from each other by large organic cations can promote exciton self-trapping and/or excited-state structural reorganization, resulting in a high photoluminescence quantum yield. As such, the co-crystallization of single/multinuclear metal-halide anions and cations is a promising approach toward building the versatile structures and interactions necessary to discover new phosphor systems. To date, however, ultralong RTP for 0D metal-halide hybrid materials remains unexploited. Facilely tunable metal and halide elements can be expected to provide a means of efficient modulation of spin–orbital coupling toward high-efficiency RTP output for practical applications. Moreover, anticipated severe restriction of the rotation and vibration of organic units due to the multiple noncovalent interactions with the surrounding metal-halide clusters could effectively facilitate resistance of the heat-quenching effect within the metal halide.

Herein, 0D metal-halide hybrids serve as a model system to spearhead the challenge of developing materials that achieve zero-TQ phosphorescence. The tetraphenylphosphine cation (Ph_4_P^+^) was selected to supply organic moieties due to its potential to obtain long-lived triplet excitons. Its large C_3_ symmetric skeleton is predicted to be relatively resistant to rotation upon heating. Furthermore, energy transfer between different energy levels can also compensate for energy loss associated with heat quenching. The optimized cluster units and halide components of the (Ph_4_P)_2_Cd_2_Br_6_ enable it to exhibit a definitive zero-TQ phosphorescence and stable RTP intensity across a wide temperature range (100–320 K), together with ultralong RTP lifetime (*τ* = 37.85 ms, *λ*_em_ = 500 nm) and high RTP quantum yield (QY_phos_ = 62.79%). This QY value marks a new record among state-of-the-art molecular long-afterglow RTP materials. Moreover, the wide tunability of excitation-dependent RTP also endows the metal halides with great potential for applications in information encoding and optical logic gates. Therefore, this work not only provides an alternative strategy to achieve wide-ranging zero-TQ luminescence, but also introduces a new perspective on designing high-efficiency and ultralong RTP based on 0D metal-halide hybrids.

## Results

### Construction of 0D metal-halide hybrids

**Fig. 1:**
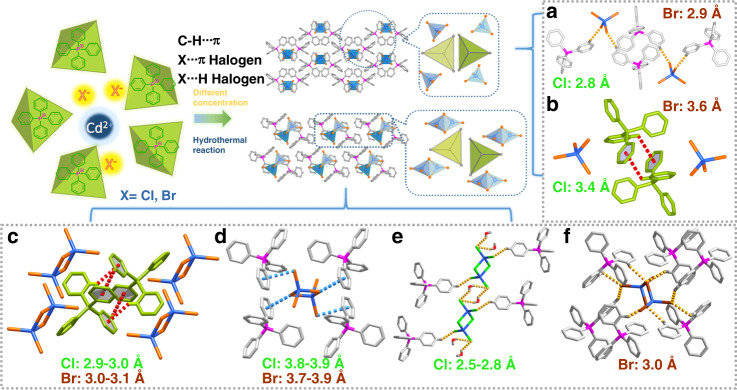
Synthetic schematic diagram and intermolecular forces. **a**, **b** (Ph_4_P)_2_CdX_4_ and **c**, **d** (Ph_4_P)_2_Cd_2_X_6_, which show the hydrogen bonds as well as C‒H···π and X···π interactions within (Ph_4_P)_2_Cd_2_Cl_6_ and (Ph_4_P)_2_Cd_2_Br_6_. **a**–**f** The orange parts refer to the halogen atoms Cl or Br, blue parts refer to the Cd atoms, the green parts refer to the Cl atoms in the figure, the brown parts refer to the Br atoms, the purple parts refer to the P atoms, and the gray parts refer to the C atoms. The tetraphenylphosphine units drawn in green refer to the cationic pairs in the crystal, echoing the green tetrahedron in the synthesis diagram.

To promote spin–orbital coupling for ultralong-lived RTP, cadmium and different halogens (X = Cl and Br) with tunable heavy atomic effects were selected to serve as the metal-halide units. Representative systems consisting of four new metal-halide organic–inorganic hybrids with 0D single-nuclear (CdX_4_^2−^) and dinuclear (Cd_2_X_6_^2−^) clusters were successfully designed and co-crystallized via a facile hydrothermal reaction (Fig. 1). Single-crystal structure analysis reveals that 0D (Ph_4_P)_2_CdX_4_ metal halides adopt a monoclinic space group *C2/c* wherein one CdX_4_^2−^ tetrahedron anion is surrounded by four Ph_4_P^+^ cations, such that CdX_4_^2−^ units are separated from each other. The Cd^…^Cd distance is 10.28 Å for CdCl_4_^2−^ (or 10.38 Å for CdBr_4_^2−^), while the P^…^P distance is 6.24 Å (or 6.36 Å for CdBr_4_^2−^). The Ph_4_P^+^ units are highly isolated, which has a prohibitive effect on the formation of H- or J-type molecular aggregation. For (Ph_4_P)_2_Cd_2_X_6_, the Cd_2_X_6_^2−^ units exhibit a co-edge metal-halide tetrahedral structure, giving rise to molecular stacking between Ph groups of two adjacent cations to produce a staggered pattern and further form an extended 1D *zig-zag* linear structure (Fig. 1b, c, e). The molecular rigidity imparted by this close arrangement and relatively high degree of condensation may restrict local structural relaxation of excited states, thereby inhibiting molecular rotation-induced nonradiative transition. Moreover, since the crystal structure appears relatively unimpacted by whether the halogen atom is Cl or Br, the single-nuclear and dinuclear hybrids in (Ph_4_P)_2_CdX_4_ (Supplementary Table 2) and (Ph_4_P)_2_Cd_2_X_6_ (Supplementary Table 3) can be regarded as isostructural systems, respectively. In both co-crystallized structures, the inorganic metal halides and organic cations exhibit a long-range ordered arrangement through cation–anion electrostatic interaction and C‒H^…^Cl (or C‒H^…^Br) hydrogen halide bonds (Fig. 1a, e, f). Under the similar structure, the differences in C‒H···π and X···π for different halogen atoms are only 0.1–0.2 Å (Fig. 1b–d). The Cl^…^H bond lengths in (Ph_4_P)_2_Cd_2_Cl_6_ are mainly 2.5–2.8 Å, while in (Ph_4_P)_2_Cd_2_Br_6_, the bond lengths are ca. 3.0 Å. It is also shown that there are water molecules in the crystal lattice of (Ph_4_P)Cd_2_Cl_6_, while not in the case of (Ph_4_P)Cd_2_Br_6_. The inclusion of solvents can be attributed to the following facts: (1) the atomic radius of the Br (0.114 nm) is larger than that of Cl (0.099 nm), i.e., when Cl replaces the position of Br, small water molecules enter the structure to fill into the cavity and form Cl^…^H bonds for structural stability; (2) the Pauling electronegativity of Cl (3.0) is stronger than that of Br (2.8), and thus it is easier for Cl binding hydrogen in water molecules to form hydrogen bonds.

### Photoluminescence spectra and room-temperature performance of metal-halide hybrids

**Fig. 2:**
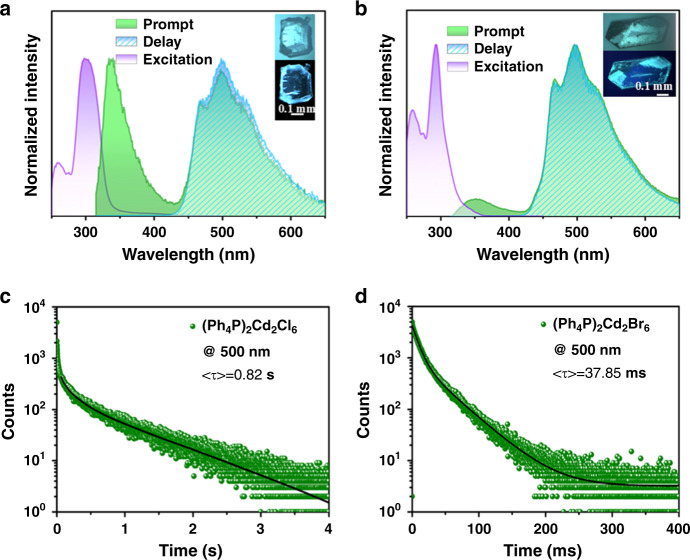
Luminescence performance of (Ph_4_P)_2_Cd_2_X_6_. Excited, prompt, and delayed PL spectra of **a** (Ph_4_P)_2_Cd_2_Cl_6_ and **b** (Ph_4_P)_2_Cd_2_Br_6_ (the delay time *t*_d_ is set to 0.1 ms). Inset pictures are photos of crystals with and without excitation. Time-resolved PL decay curve of **c** (Ph_4_P)_2_Cd_2_Cl_6_ and **d** (Ph_4_P)_2_Cd_2_Br_6_ (*λ*_ex_ = 300 nm).

The photophysical properties of the as-prepared metal halides were investigated at ambient conditions (Fig. 2 and Supplementary Fig. 1). The prompt PL spectra (Fig. 2a, b) exhibit a typical two-band emission, with one in the ultraviolet region (*λ*_em_ ≈ 350 nm) and the other corresponding to green wavelengths and possessing three major peaks around 470, 500, and 540 nm. The delayed PL spectra of (Ph_4_P)_2_Cd_2_X_6_ overlap nearly perfectly with the long-wavelength regions of the prompt one, suggesting that the photoluminescence of (Ph_4_P)_2_Cd_2_X_6_ feature both fluorescence and phosphorescence two-band characteristics. To identify the luminescent contribution of these materials, we measured the spectra of their corresponding organic ionic cocrystals (Ph_4_PCl and Ph_4_PBr) and four metal halides dissolved in N,N-dimethylformamide, which show only a sharp luminescence peak around 320 nm (Supplementary Fig. 2). In contrast, the crystalline forms of Ph_4_PCl and Ph_4_PBr show double emission around 350 and 500 nm (Supplementary Fig. 3a, c). The luminescence at 350 nm is consistent with the red-shifted singlet emission commonly observed in organic solid-state materials compared to their solutions^43^. The emission at 500 nm can be attributed to typical crystallization-induced RTP behavior. The RTP QY_phos_ values for the Ph_4_PCl and Ph_4_PBr are 8.02% and 21.77%, respectively (Supplementary Table 4). The relative high photoluminescence for Ph_4_PBr is related to the increased intermolecular distance as the change of anions from Cl to Br, and consequently weaker intermolecular electronic coupling results in the decrease of aggregation-induced quenching. Moreover, Br atom has a stronger spin–orbit coupling effect relative to that of Cl atom, which promotes the intersystem crossing rate, and thus is more inclined to RTP emission.

Together with the observations above and obvious different emissions from pristine CdX_2_ (Supplementary Fig. 4), the PL nature of (Ph_4_P)_2_Cd_2_X_6_ is thus originally derived from its organic Ph_4_P^+^ unit. It is also noted that the RTP emission of (Ph_4_P)_2_Cd_2_Br_6_ is largely dominant over that of (Ph_4_P)_2_Cd_2_Cl_6_ in the PL spectra, which can be ascribed to the stronger spin–orbital coupling of Cd_2_Br_6_^2−^ units compared to those of Cd_2_Cl_6_^2−^, and results in the acceleration of intersystem crossing processes. In addition, the relationship between the prompt and delayed PL spectra clearly varies depending on the identity of (Ph_4_P)_2_CdX_4_:(Ph_4_P)_2_CdCl_4_ exhibits nearly complete overlapping of its delayed mode and prompt spectra, while (Ph_4_P)_2_CdBr_4_ exhibits strong fluorescence but very weak RTP output. This behavior may be related to the RTP emission quenching through the triplet–triplet annihilation process^31^. The luminescence performance of (Ph_4_P)_2_Cd_2_X_6_ is generally better than (Ph_4_P)_2_CdX_4_, which can be attributed to that the distance between organic emissive molecules is closer in (Ph_4_P)_2_CdX_4_ (Supplementary Table 5), and thus the energy is easier to be dissipated in the form of nonradiative transitions. Therefore, introducing different metal-halide clusters is an effective means of adjusting the singlet–triplet energy distribution and photoluminescence. To detect the role of different metals in the RTP emission, we have further synthesized Pb halide-based system, (Ph_4_P)_2_Pb_3_Cl_8_ as a control example. It shows much shorter luminescence lifetime of 9.56 μs at 500 nm excited at 300 nm. The structure also exhibits that the distance between organic molecules becomes larger and the interaction becomes smaller (Supplementary Fig. 5). This indicates that both the metal halide with moderate heavy-atom effect and suitable packing fashion could lead to different long-lived RTP emission.

To better understand the excitation-state properties, the time-resolved PL spectra of the metal-halide hybrids were measured at room temperature. The longest RTP lifetime of 0.82 s occurs at (Ph_4_P)_2_Cd_2_Cl_6_, with the corresponding QY_phos_ of 23.49%, while the (Ph_4_P)_2_Cd_2_Br_6_ exhibits the RTP lifetime of 37.85 ms and QY_phos_ of 62.79% (Fig. 2c, d). These results are consistent with the emissive characteristics of metal-free Ph_4_PCl and Ph_4_PBr cocrystals (Supplementary Fig. 3), namely the long-lived phosphorescence but weak intensity of the former versus the shorter phosphorescence lifetime but enhanced RTP of the latter. Such behaviors are rooted in the inherently competitive relationship between RTP QY_phos_ and lifetime. Notably, the QY_phos_ value reported in this study for (Ph_4_P)_2_Cd_2_Br_6_ exceeds those of other as-reported molecule-based RTP systems (Supplementary Table 6). The demonstrated combination of high RTP QY_phos_ and ultralong lifetime is an important advancement in the development of high-quality solid-state light-emitting applications.

To obtain insight into the influence of water molecules on RTP of (Ph_4_P)_2_Cd_2_Cl_6_, we further performed a control experiment after dehydration by heating. The thermogravimetric analysis (TGA, Supplementary Fig. 6) curve shows that the first step weight loss occurs at around 110 °C (384 K), which corresponds to the loss of water content (1.59%) determined by single-crystal XRD analysis. Thus, we have heated the sample at 110 °C (384 K) for an hour to remove the water and then cooled down to room temperature. It was observed that the RTP intensity of the dehydration system has slightly decreased in the range of 450–550 nm (Supplementary Fig. 7a), and the RTP lifetime has also decreased from 0.82 s to 0.71 s compared with the pristine form (Supplementary Fig. 7b), indicating that the inclusion of water molecules could stabilize the RTP to some extent based on the hydrogen-bonding interactions, and similar behavior also occurs for some other molecular cocrystal and carbon dot systems^44,45^. Moreover, to study the stability of the water-containing phase at and above room temperature under illumination, we selected the typical temperatures (300 K and 320 K) to detect the photostability. It was observed that the RTP intensity can be readily repeated during the heating–cooling process at two typical temperatures (Supplementary Fig. 7c), suggesting that RTP emission of the water-containing phase is stable at room temperature. Moreover, we performed the alternate illumination on the sample at typical excitation 254 and 365 nm (Supplementary Fig. 7d), and the luminescent intensity is also repeatable under different illumination, confirming the photostability of the sample upon irradiation.

### Temperature-resistant properties in metal halides

**Fig. 3:**
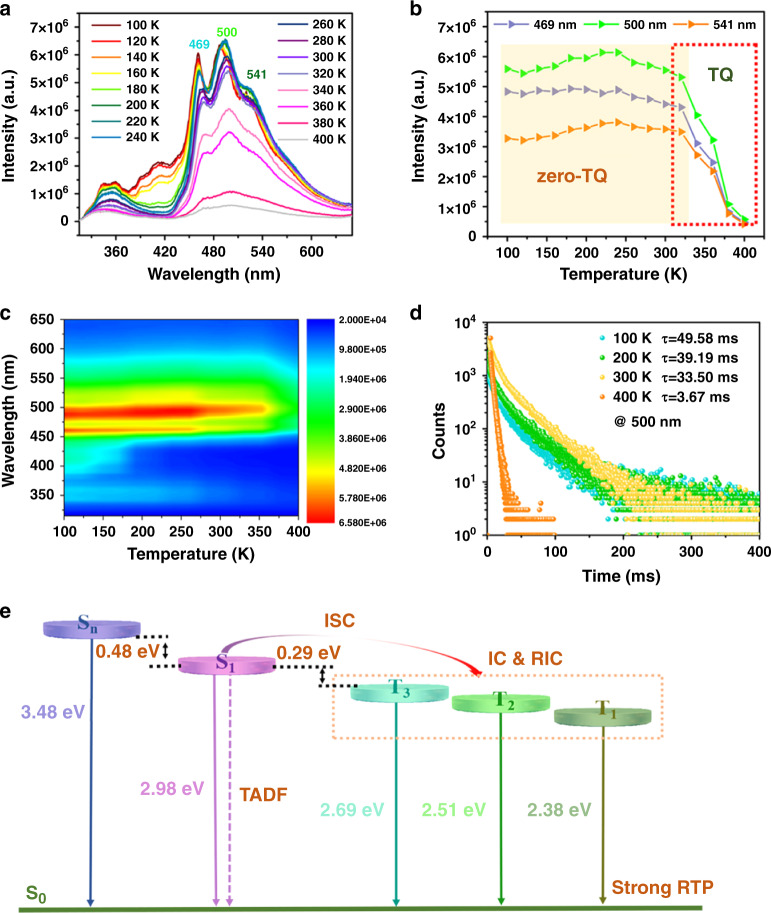
Emission spectra of (Ph_4_P)_2_Cd_2_Br_6_ under different temperatures and the analysis. Solid-state emission spectra of **a** (Ph_4_P)_2_Cd_2_Br_6_ at different temperatures between 100 and 400 K upon excitation at 300 nm. **b** Temperature-dependent intensity of emission (469 nm, 500 nm, 541 nm) from 100 to 400 K. **c** Two-dimensional map of the PL spectra of (Ph_4_P)_2_Cd_2_Br_6_ under the prompt mode at different temperatures ranging from 100 to 400 K. **d** Time-resolved PL decay curve of (Ph_4_P)_2_Cd_2_Br_6_ at different temperatures. **e** Energy levels of different states (calculated with phosphorescence peak at 100 K).

To probe the thermally luminescent properties of the 0D metal-halide hybrids, the temperature-dependent emission measurements were performed on the (Ph_4_P)_2_Cd_2_Br_6_, which exhibited both high RTP QY and ultralong lifetime. The PL emission spectra (Fig. 3a) reveal three prominent phosphorescence bands with relatively close triplet states (T_1_, T_2_, and T_3_), which undergo a slight redshift as temperature increases from 100 to 400 K^46^. The phosphorescence intensity of (Ph_4_P)_2_Cd_2_Br_6_ is particularly stable over a wider temperature range (100–320 K), with the T_1_ and T_3_ band at 320 K maintaining intensities 95% of those at 100 K (Fig. 3b, c). The emission intensity finally begins to decline at ~340 K. Moreover, increasing temperature evokes only a slight decrease of the lifetimes for the (Ph_4_P)_2_Cd_2_Br_6_ from 49.58 ms (100 K) to 33.50 ms (300 K) (Fig. 3d and Supplementary Table 7), suggesting that it can maintain its ultralong phosphorescent nature across a broad temperature range (>200 K). Therefore, the (Ph_4_P)_2_Cd_2_Br_6_ can be regarded as a new type of zero-TQ phosphorescent materials with ultralong lifetime. To the best of our knowledge, although the zero TQ luminescent materials based on an inorganic compound and metal complex have been developed quite recently^23–27^, thermally resistant systems involving ultralong RTP have not yet been reported, particularly for metal-halide materials.

**Fig. 4:**
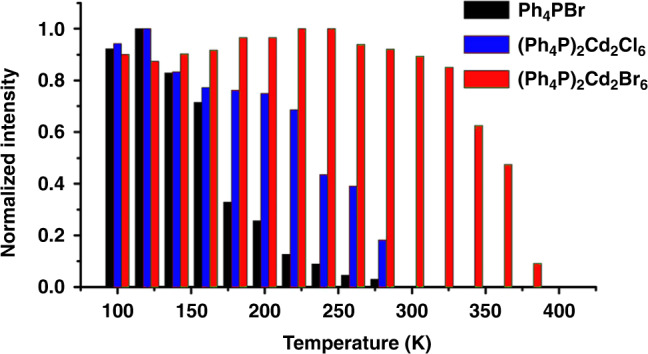
Comparison of temperature influence on room-temperature performance. Temperature-dependent intensity of emission (at 500 nm) (Ph_4_PBr: 100–300 K; (Ph_4_P)_2_Cd_2_Cl_6_: 100–300 K; (Ph_4_P)_2_Cd_2_Br_6_: 100–400 K).

As a counterpart to (Ph_4_P)_2_Cd_2_Br_6_, evaluation of the TQ effect in metal-free ionic cocrystal Ph_4_PBr (Supplementary Fig. 8) indicates that the luminous intensity systematically decreases as the temperature increases from 140 to 300 K. This divergence between the temperature-dependent behaviors of the Ph_4_PBr and (Ph_4_P)_2_Cd_2_Br_6_ verifies that the metal-halide units play a key role in inhibiting the TQ effect of phosphorescence over a wide temperature range. It is further observed that increasing temperature provokes a more rapid decrease to the phosphorescence peak intensity of (Ph_4_P)_2_Cd_2_Cl_6_ than to that of the (Ph_4_P)_2_Cd_2_Br_6_ (Fig. 4), with the former exhibiting about 20% emission loss from 100 to 260 K. This difference, which is attributed to the stronger heavy-atom effect in the Cd_2_Br_6_^2−^ clusters than in Cd_2_Cl_6_^2−^, is indicative of the suitability of Cd_2_Br_6_^2−^ for achieving zero-TQ phosphorescence. Moreover, comparisons of the crystal parameters of (Ph_4_P)_2_Cd_2_Br_6_ at 100 K and room temperature (Supplementary Tables 8–11) reveal that increasing temperature evokes a slight increase of the Cd‒Br distance of the Cd_2_Br_6_^2−^ units (Supplementary Table 11), while the C‒P distance of the Ph_4_P^+^ is nearly unaffected, resulting in ~3% expansion of the crystal lattices and slight structural torsion of the Ph_4_P^+^ units. It is reported that the temperature-induced structural torsion or phase change may facilitate TQ resistance in pure inorganic solids^23–27^. From a structural perspective, the temperature effect on the Cd_2_Br_6_^2−^ clusters and expansion of the crystal lattice may even reduce the thermally induced molecular vibrations of Ph_4_P^+^ to some extent, which is also confirmed by theoretical simulation below.

It is observed that initial increases in intensities of the major phosphorescent emission (T_2_) and the shoulder band (T_1_) occur as the temperature increases from 100 to 240 K (Fig. 3b), accompanied by a slight decrease of the high-energy T_3_ band. These observations can be attributed to the thermally active phosphorescent enhancement, wherein thermal energy assists dynamic energy transfer by promoting the capturing and releasing of triplet excitons among three closely positioned energy levels^46^. Moreover, it is noted that at low temperature, additional luminescence at 410 nm for (Ph_4_P)_2_Cd_2_Br_6_ (Supplementary Fig. 9) and 421 nm for (Ph_4_P)_2_Cd_2_Cl_6_ (Supplementary Figs. 10 and 11) appears, although these signals decrease continuously with increasing temperature. Once the emission intensity at 410 or 421 nm decreases to almost zero, the phosphorescence starts to decrease. Thus, it can be reasonably deduced that the two weak bands at 410 and 421 nm largely comprise the energy loss observed upon increasing temperature. To figure out the contribution of the peaks at 410 and 421 nm, the photophysical properties of the pristine Ph_4_PCl and Ph_4_PBr cocrystals were analyzed and found to possess major emission peaks at 410 and 466 nm, respectively, upon excitation at 365 nm (Supplementary Fig. 3e, f). The delayed spectrum evidences that Ph_4_PCl exhibits very weak luminescence with two peaks at 410 and 500 nm, while that of Ph_4_PBr reveals loss of long-lived emission. Moreover, the emission of Ph_4_PBr at about the 400–420-nm position can be obtained in the monomer solution state, indicating that its position can be attributed to a singlet state.

The PL decay curves of (Ph_4_P)_2_Cd_2_Br_6_ show that the lifetime at ca. 410 nm increases with temperature from 5.28 (100 K) to 42.49 μs (300 K), revealing that this position is characterized by a certain degree of thermally activated delayed fluorescence (TADF) (Supplementary Figs. 9b and 12). Therefore, the reduced TADF strength and the slow decay of phosphorescent intensity with increasing temperature indicates that the adjacent singlet state (S_1_) serves as an intermediate energy level that captures energy and transmits it to the triplet-energy state, thereby reducing the nonradiative transition, maintaining phosphorescence intensity, and increasing quantum yield. The adjacent singlet TADF prevents thermally induced energy loss via phosphorescence to some extent. Increasing the temperature cannot fully compensate for the phosphorescence attenuation caused by TQ, so that as the TADF gradually decays to zero, the phosphorescence begins to undergo a distinct decrease. Based on the S_1_ and T_3_ excited-state levels of (Ph_4_P)_2_Cd_2_Br_6_ (S_1_: 2.98 eV, 416 nm; T_3_: 2.69 eV, 461 nm) and (Ph_4_P)_2_Cd_2_Cl_6_ (S_1_: 2.95 eV, 421 nm; T_3_: 2.67 eV, 464 nm), their energy gaps (ΔE_ST_) between singlet and triplet states are very similar, namely 0.29 and 0.28 eV, respectively (Fig. 3e). Comparing the temperature-dependent luminescence behaviors of Ph_4_PBr, (Ph_4_P)_2_Cd_2_Cl_6_, and (Ph_4_P)_2_Cd_2_Br_6_, Fig. 4 illustrates that both the heavy-atom effect and thermally active triplet-energy transfer are important for achieving effective temperature-resistant phosphorescence.

### Hirshfeld surface and fingerprint plot analyses

To evaluate the detailed intermolecular interactions and molecular rigidification structure of the zero-TQ (Ph_4_P)_2_Cd_2_Br_6_ from a quantitative view, we conducted the Hirshfeld surface and fingerprint plot analyses. The Hirshfeld surface of (Ph_4_P)_2_Cd_2_Br_6_ is mapped over d_norm_, shape index, and curvedness. The d_norm_ surface is the normalized function of d_i_ and d_e_ (Supplementary Figs. 13 and 14), with white-, red-, and blue-colored surfaces. The white surface indicates those contacts with distances equal to the sum of the van der Waals (vdW) distance; red and blue indicate shorter contact (<vdW distance) and the longer contact (>vdW distance), respectively. The deep red color indicates the presence of Br···H hydrogen-bonding contact, while blue and white color spots indicate the presence of other close contacts, such as H···H, Cd···H, Cd···C, Br···C, and C···H. The two-dimensional fingerprint plot analysis for all contacts of (Ph_4_P)_2_Cd_2_Br_6_ are shown in Supplementary Figs. 13g and  14g. The relative contributions to the Hirshfeld surface area for each type of intermolecular contact are illustrated in Supplementary Figs. 13 and  14. As for the large number of white regions that still exist in the d_norm_ surfaces, it indicates that there is also weak and distant contact between molecules.

The void space within crystals is considered as the free volume. The low free volume usually corresponds to high molecular rigidification and confinement. The fractional free volume (FFV) can be defined as

1 $${\mathrm{FFV}} = \frac{{V{\mathrm{f}}}}{{V{\mathrm{f}} + V{\mathrm{o}}}} \times 100\% ,$$

where *V*_f_ is the free volume and *V*_o_ is the volume occupied by the materials. It is observed that the FFV for (Ph_4_P)_2_Cd_2_Br_6_ at different temperatures (Supplementary Table 12) is very low (<10%)^47^, suggesting high rigidification with less molecular vibrational and rotational motions. In detail, the FFV is 9.30% at 289 K, slightly larger than that at 100 K (7.82%). The system does get enhanced molecular thermal motion under heating conditions, with a slightly looser structure and a slightly less interaction. Comparison results based on single-crystal XRD test (Supplementary Fig. 15f) show that although the vibration becomes larger at high temperature, the equilibrium position does not change obviously.

### Molecular dynamics simulation and density functional theory (DFT) calculations

**Fig. 5:**
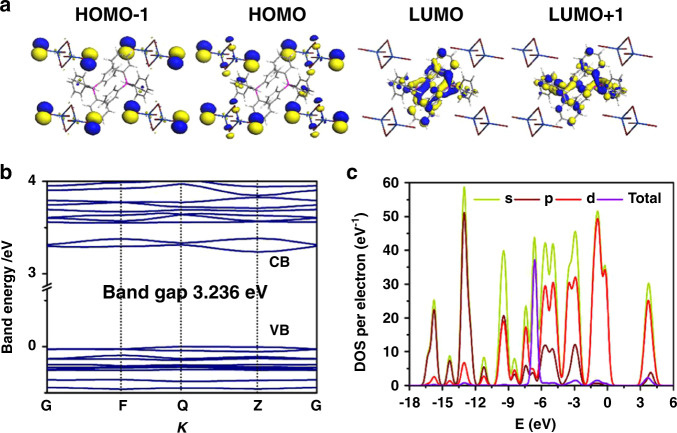
Density functional theory (DFT) calculations of (Ph_4_P)_2_Cd_2_Br_6_. The calculated molecular orbitals **a** HOMO-1, HOMO, LUMO, and LUMO + 1. Band structures around the Fermi energy level **b** and the total/partial electronic density of states **c** for (Ph_4_P)_2_Cd_2_Br_6_. G (0, 0, 0), F (0, 1/2, 0), Q (1/2, 0, 0), and Z (0, 0, 1/2) are the selected reciprocal points in the first Brillouin zone (BZ).

To better understand how structural factors contribute to the zero-TQ phosphorescence of (Ph_4_P)_2_Cd_2_Br_6_, molecular dynamics simulations were performed at typical thermodynamic temperatures of 100 and 290 K (which closely correspond to the experimental conditions above). It is observed that the Cd_2_Br_6_^2−^ is characterized by a much more prominent fluctuation of its torsion angle than the Ph_4_P^+^ cations are (Supplementary Fig. 15a–d), indicating that the Cd_2_Br_6_^2−^ clusters serve as the structural relaxation units to reduce the thermal effect on Ph_4_P^+^. The average calculated Cd‒Br and P‒P distances are also consistent with the trend obtained from single-crystal structures at high/low temperatures (Supplementary Fig. 15e). Furthermore, periodic DFT calculations were performed to gain insight into band structure, density of states (DOS), and electron-density distributions. Total and partial electronic densities of states evidence that the (Ph_4_P)_2_Cd_2_Br_6_ system has a small bandgap of 3.236 eV (Fig. 5c), and this value is close to the experimental result calculated from the UV–vis absorption edge (3.28 eV) (Supplementary Fig. 16). Band structure calculations indicate that both the conduction band (CB) minimum and the valence band (VB) maximum are localized at the Z point, confirming that it is a direct bandgap semiconductor, in agreement with the high solid-state QY_phos_ emission measured experimentally (Fig. 5c). Partial electronic density of states (PDOS) reveals that the CB and VB are derived from the *p* orbitals of C and P atoms (Supplementary Fig. 17b). The highest occupied molecular orbital (HOMO) predominantly consists of *p* orbitals of Br in Cd_2_Br_6_^2−^ clusters and adjacent π orbitals of benzene in Ph_4_P^+^ units, corresponding to halogen–π interactions (Fig. 1d). The lowest unoccupied molecular orbital (LUMO) is solely localized on the *p* orbitals of C atoms in four benzene groups in whole organic Ph_4_P^+^. Similar results are calculated for (Ph_4_P)_2_Cd_2_Cl_6_ (Supplementary Figs. 17a and  18). This suggests that potential energy/electronic transfer between Cd_2_Br_6_^2−^ and Ph_4_P^+^, along with the strong heavy-atom effect, promotes triplet excitons (Fig. 5a, b). Combining experimental and theoretical results suggests that the zero-TQ phosphorescence of (Ph_4_P)_2_Cd_2_Br_6_ can be attributed to the following factors: (1) from an energy perspective, the heavy-atom effect of Cd_2_Br_6_^2−^ with strong spin–orbital coupling facilitates the ultralong phosphorescence dominating the overall PL process, while weak emission from high-energy singlet excitons serves as an energy buffer layer to reduce the TQ effect on triplet excitons. (2) The proximity of the triplet-energy levels leads to efficient energy transfer between different triplet states, resulting in enhanced thermally active and ultralong-lived phosphorescence across a wide temperature range. (3) From a structural perspective, the thermally induced fluctuation of Cd_2_Br_6_^2−^ clusters could act as a structural buffer layer to decrease thermal effects associated with the structural relaxation of Ph_4_P^+^ chromophores (Fig. 3 and Supplementary Fig. 9).

### Excitation-dependent RTP and Morse information encryption applications

**Fig. 6:**
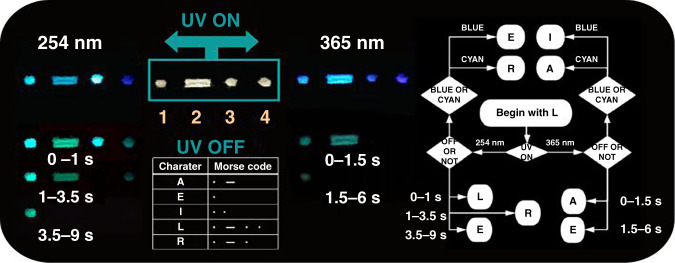
Schematic diagram of the information encryption and decryption processes. (1) (Ph_4_P)_2_Cd_2_Cl_6_, (2) (Ph_4_P)_2_CdCl_4_, (3) (Ph_4_P)_2_Cd_2_Br_6_, and (4) (Ph_4_P)_2_CdBr_4_.

Due to the multiple energy levels indicated by the experimental and calculated results, the four 0D metal halides display excitation-dependent RTP properties, wherein emission band and colors are typically tunable by excitation at 254 and 365 nm (Supplementary Figs. 19 and  20). These properties make the four 0D metal halides suitable for applications, such as information encoding and anti-counterfeiting. To demonstrate this strategy, we have developed a coding method based on the Morse code to carry out multiple information storage, since the Morse code “dot” or “dash” can be facilely designed. Upon excitation at different wavelengths, different emissive colors can be obtained from (Ph_4_P)_2_Cd_2_Cl_6_, (Ph_4_P)_2_CdCl_4_, (Ph_4_P)_2_Cd_2_Br_6_, and (Ph_4_P)_2_CdBr_4_ (Supplementary Figs. 19 and  20). Figure 6 shows that the four materials in daylight are distributed on the same black background, showing a white powder state, so the code character defined as “L” for the normal Morse password is obtained. When applying the excitation light source, we can decode different emission colors displayed by the samples. Furthermore, when the light source is turned off after excitation, we can decode it with different afterglow time of different materials. This becomes programmable: for example, if we choose excitation at 254 nm, after closing the excitation condition for 3 s, the resulting character is decoded as “R”. Like this manner and process, precise application and removal of the excitation light source can produce different Morse code patterns that correspond to different characters (defined in this work as A, E, I, L, and R). Thus, the only state in daylight can be split into five different states under UV excitation with different wavelengths. Although this procedure allows for only five different states, it is conceivable that careful selection of the excitation light source, setting of the information that is encrypted by the responding emission color, and controlled duration in the range of 1–9 s of exposure to excitation could increase the variety of data that can be stored. This encryption method is strongly secure since decryption of the data would require precise control of experimental conditions (Fig. 6).

## Discussion

In summary, new 0D metal-halide organic–inorganic hybrid materials exhibit rare zero-TQ ultralong phosphorescence over a wide temperature range (above 220 K). Unlike the well-known mechanisms of zero-TQ luminescence (such as structural torsion and phase change) and long-lived RTP (such as molecular rigidification), this work illustrates weak TADF bands that can serve as energy buffer layers to compensate the phosphorescence loss upon increasing temperature, and thus enables a new route toward achieving zero-TQ visible luminescence. Moreover, the (Ph_4_P)_2_Cd_2_Br_6_ system exhibits high QY_phos_ of 62.79%, which may stand for a new record in ultralong RTP materials, to the best of our knowledge. Furthermore, by exploiting the interesting excitation-dependent RTP of four metal halides, different combinations of the hybrids can be used for Morse code encryption and time-resolved information logic gates. Therefore, this work demonstrates the example of organic–inorganic hybrids with both zero-TQ emission and very high solid-sate RTP QY. Facile design of 0D metal-halide hybrids can be expected to provide new ways to develop materials with high-efficiency ultralong RTP for thermally resistant luminescence and information-encoding applications. Further work is still underway to explore the thermally stable phosphorescence of the materials above room temperature.

## Methods

### Materials and reagents

All the reagents (tetraphenylphosphonium chloride, tetraphenylphosphonium bromide, cadmium chloride, cadmium bromide, hydrochloric acid, and hydrobromic acid) were purchased from Sigma Chemistry Co. Ltd. and used without further purification. Distilled water is prepared in our lab.

### Synthesis of metal-halide hybrids

The crystalline (Ph_4_P)_2_CdX_4_ samples were prepared via the hydrothermal method. A mixture of CdCl_2_·4H_2_O or CdBr_2_·4H_2_O (0.6 mmol) with tetraphenylphosphonium chloride (0.6 mmol, 0.24 g) or tetraphenylphosphonium bromide (0.6 mmol, 0.25 g) and 8 mL of H_2_O was stirred for 10 min. The mixture was then transferred to a Teflon reactor (23 mL) and heated at 160 °C for 24 h. After that, the mixture was cooled at a rate of 5 °C h^−1^ to room temperature. The colorless transparent crystals of (Ph_4_P)_2_CdX_4_ were washed by ethanol (3 × 10 mL). The crystalline (Ph_4_P)_2_Cd_2_X_6_ samples were prepared via the hydrothermal method. A mixture of CdCl_2_·4H_2_O or CdBr_2_·4H_2_O (1.2 mmol) with tetraphenylphosphonium chloride (0.6 mmol, 0.24 g) or tetraphenylphosphonium bromide (0.6 mmol, 0.25 g) and 8 mL of H_2_O was stirred for 10 min. The mixture was then transferred to a Teflon reactor (23 mL) and heated at 160 °C for 24 h. After that, the mixture was cooled at a rate of 5 °C h^−1^ to room temperature. The colorless transparent crystals of (Ph_4_P)_2_Cd_2_X_6_ were washed by ethanol (3 × 10 mL).

### Structural and morphology characterization

Single-crystal X-ray diffraction data of these samples were collected on an Oxford Diffraction SuperNova area-detector diffractometer using mirror optics monochromated Cu Kα radiation at room temperature. UV–vis absorption spectra were performed on Shimadzu UV-3600 spectrophotometer at room temperature. PL microscope images of crystals were taken under OLYMPUS IX71 fluorescence microscope. All the relevant PL tests, including fluorescence and phosphorescence and time-resolved lifetime, were conducted on an Edinburgh FLS980 fluorescence spectrometer. The PLQY of the crystals was determined by using a Teflon-lined integrating sphere (F-M101, Edinburgh, diameter: 150 mm and weight: 2 kg) accessory in FLS980 fluorescence spectrometer.

### Molecular dynamics (MD) simulations

The calculations were performed by classical molecular dynamic simulation method employing a universal field. Charge equilibration (QEq) method^48^ was used to calculate atomic charges. In potential energy calculations, the long-range Coulomb interactions between partial charges were computed by the Ewald summation technique, and a “spline cutoff” method was used to calculate the van der Waals interaction. After energy minimization was applied on the initial model, MD simulations were performed in isothermal–isobaric (NPT) ensemble with the typical thermodynamic temperatures of 100 and 290 K (corresponding to the typical measurement in the experiment) and the pressure of 0.1 MPa (~1 atm). The Andersen method^49^ and Berendsen method^50^ were used to control temperature and pressure, respectively. The total simulation time was 250 ps with the simulation time step of 1 fs. The result shows that the system reached equilibrium with lattice parameters and total potential energy fluctuating around a constant value within the first 50 ps, so the dynamic trajectories were recorded every 20 fs in the remaining 200 ps in order to analyze the ensemble average values. The simulations were performed using Forcite module in Material Studio software package^51^.

### Electronic structure calculations

The calculations were performed with the periodic DFT method by using Dmol3 module in Material Studio software package^51–53^. The initial configurations were fully optimized by the Perdew–Wang (PW91)^54^ generalized gradient approximation (GGA) method with the double numerical basis sets plus polarization function (DNP). The core electrons of metals were treated by effective core potentials (ECP). The self-consistent field (SCF) converged criterion was within 1.0 × 10^−5^ hartree per atom and the converged criterion of the structure optimization was 1.0 × 10^−3^ hartree per Bohr. The Brillouin zone was sampled by 1 × 1 × 1 k points, and test calculations reveal that the increase in k points does not affect the results.

### Hirshfeld surfaces and 2D fingerprint plot calculation

The Hirshfeld surfaces and 2D fingerprint plots were calculated by using Crystal Explorer 17.5^55^.

## Supplementary information

Supplementary Information

## Data Availability

Data supporting the findings of this paper are available from the corresponding authors upon reasonable request. The crystallographic coordinates for structures reported in this paper have been deposited at the Cambridge Crystallographic Data Centre (CCDC) under deposited number CCDC: 1971490 ((Ph_4_P)_2_CdCl_4_), 1971488 ((Ph_4_P)_2_Cd_2_Cl_6_), 1971489 ((Ph_4_P)_2_CdBr_4_), 1971491 ((Ph_4_P)_2_Cd_2_Br_6_—100 K), and 1971487 ((Ph_4_P)_2_Cd_2_Br_6_—289 K).
