# Supplementary material for: Wide range zero-thermal-quenching ultralong phosphorescence from zero-dimensional metal halide hybrids
Source: Nat Commun. 2020 Sep 16;11:4649. doi: 10.1038/s41467-020-18482-w (PMC7494901; doi:10.1038/s41467-020-18482-w)
Supplement: Supplementary file 1 — Supplementary Information [file 41467_2020_18482_MOESM1_ESM.pdf]

Wide range zero-thermal-quenching ultralong phosphorescence from zero-dimensional metal halide hybrids

Liu *et al.*

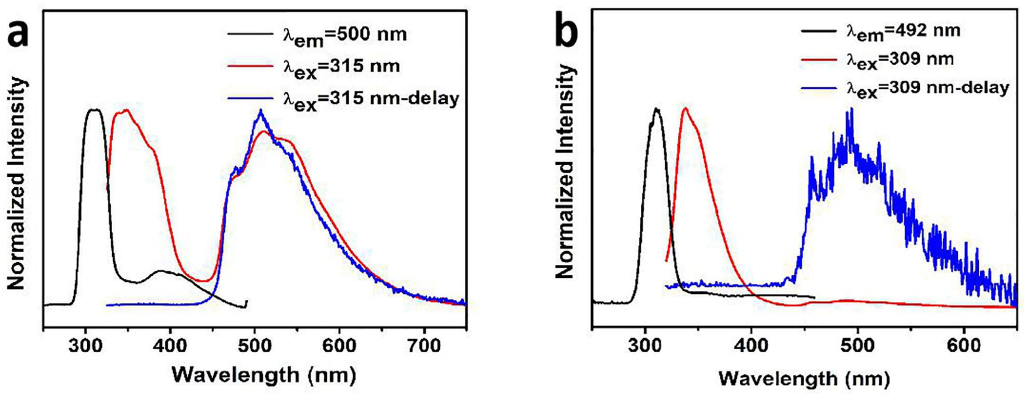

Supplementary Figure 1 | PL spectra of $(Ph_4P)_2CdCl_4$ (a) and $(Ph_4P)_2CdBr_4$ (b) at room temperature.

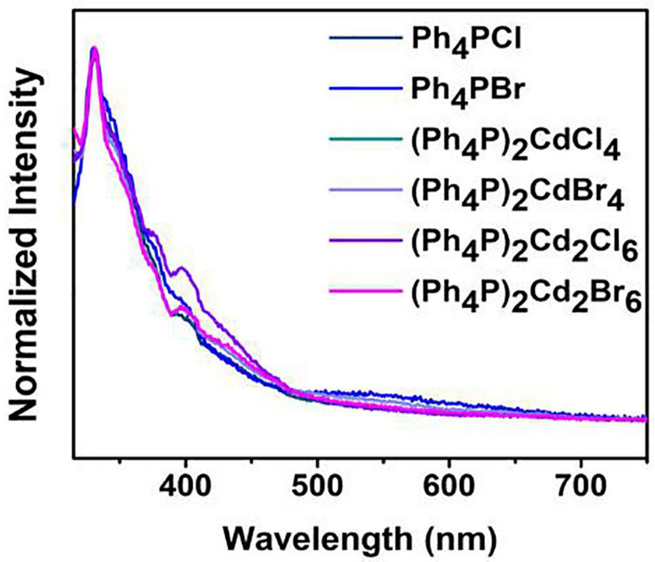

Supplementary Figure 2 | PL spectra of Ph_4PCl, Ph_4PBr, $(Ph_4P)_2CdCl_4$, $(Ph_4P)_2Cd_2Cl_6$, $(Ph_4P)_2CdBr_4$, $(Ph_4P)_2Cd_2Br_6$ dissolved in N,N-dimethylformamide (DMF) at room temperature. (λ_{ex}=300 nm)

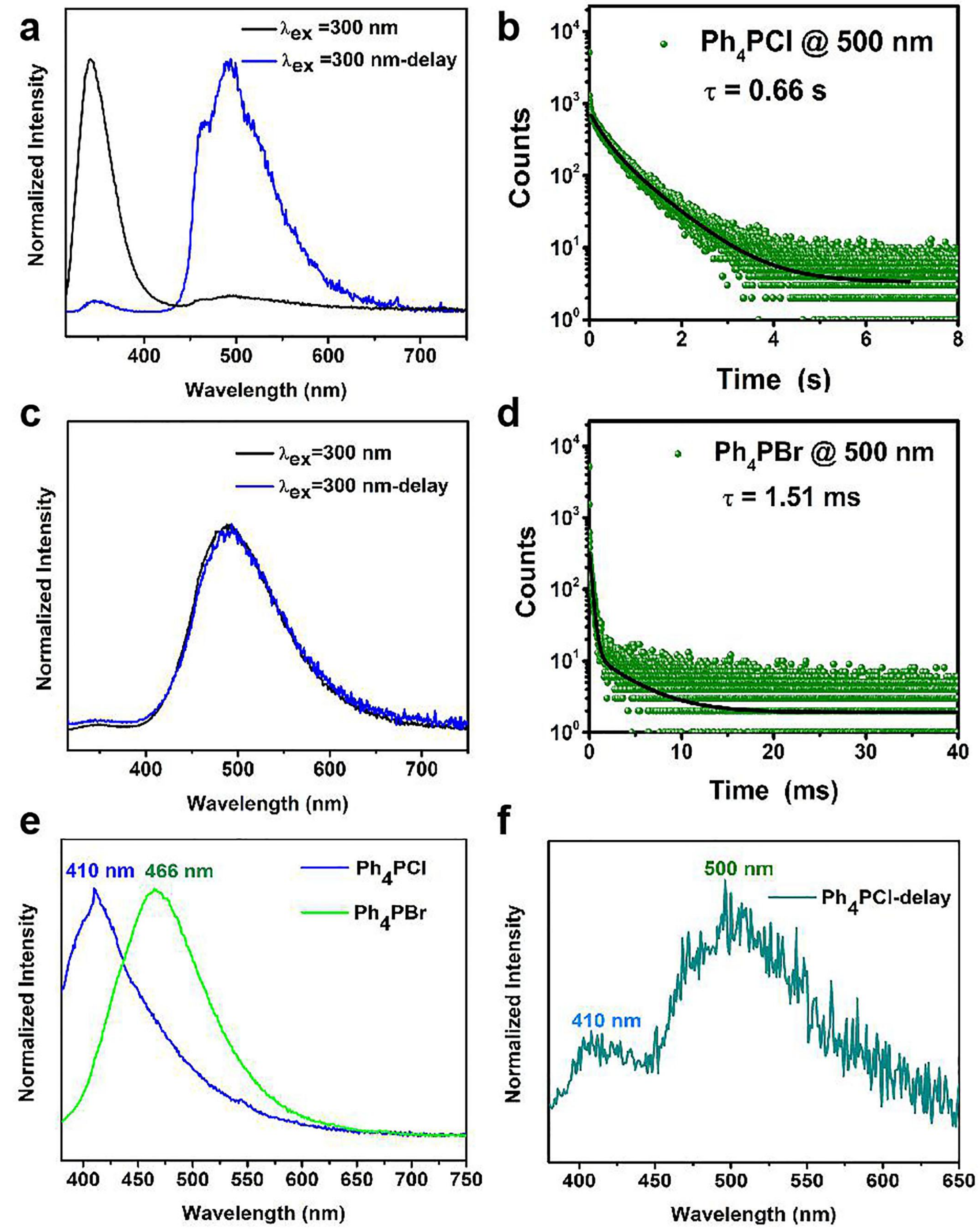

Supplementary Figure 3 | Prompt and delayed PL spectra of Ph_4PCl (a) and Ph_4PBr (c); Time-resolved PL-decay profiles of Ph_4PCl (b) and Ph_4PBr (d). ((a), (b), (c) and (d) are excited at 300 nm; (e) and (f) are excited at 365 nm)

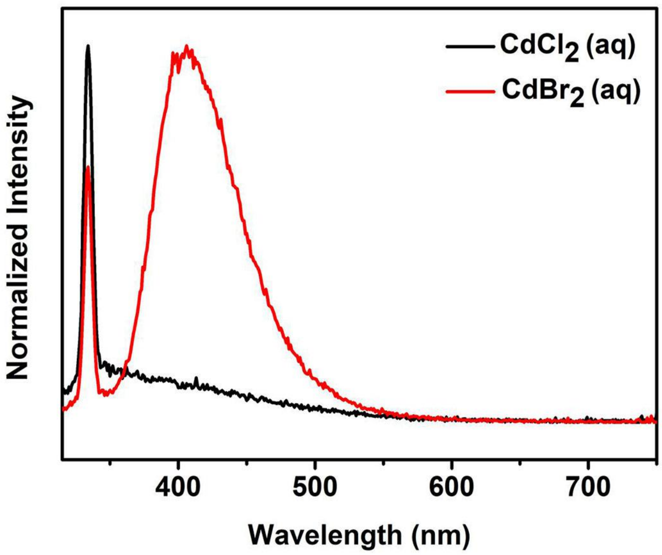

Supplementary Figure 4 | Emission spectra of $CdCl_2$ and $CdBr_2$ aqueous solutions (10^{-5} mol/L). (λ_{ex}=300 nm)

There is no visible photoemission of $CdCl_2$ but weak emission at ca. 410 nm for $CdBr_2$ in their solution samples (10^{-5} mol/L). The emission position is far from that in the inorganic-organic hybrids. The solid-state emission of the films coated from the solutions cannot be detected, and the luminescence from these samples is unobserved by the naked eye under UV light.

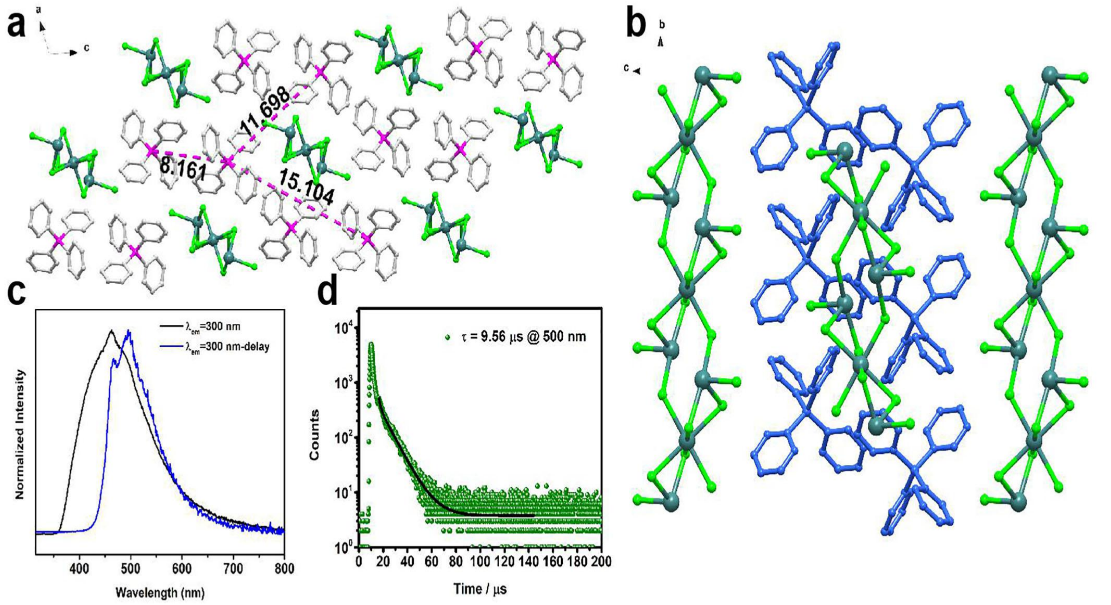

Supplementary Figure 5 | $(Ph_4P)_2Pb_3Cl_8$ (a) structure views along the b axis with distances between Ph_4P^+ cations and between dimers (Å); (b) structure views along the a axis; (c) prompt and delayed PL spectra and (d) time-resolved PL decay curve.The protons are removed for the sake of clarity. (The green sphere represents the Cl atom while the teal sphere represents the lead atom.)

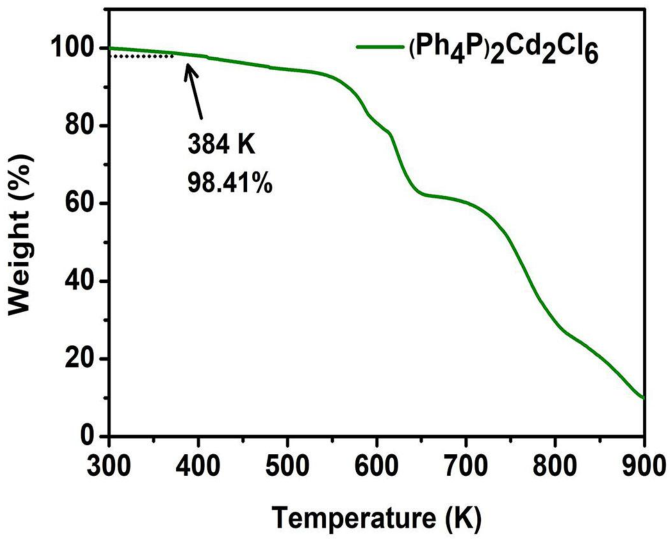

Supplementary Figure 6 | The TGA curve of $(Ph_4P)_2Cd_2Cl_6$.

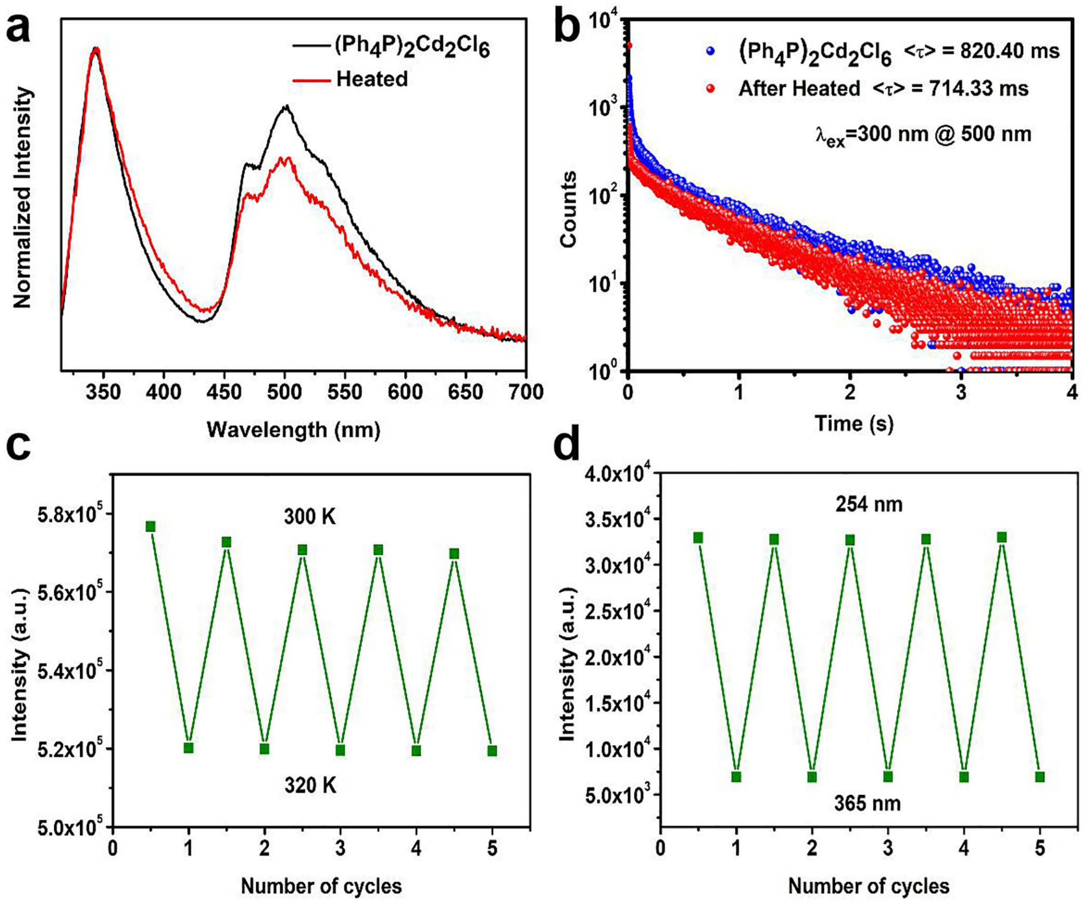

Supplementary Figure 7 | Luminescence spectra (a) and lifetimes (b) of $(Ph_4P)_2Cd_2Cl_6$ before and after heated at 110°C for one hour then cooling down; (c) reversible variation of the phosphorescence intensty of $(Ph_4P)_2Cd_2Cl_6$ at around room temperature (300 K and 320 K); (d) reversible variation of the phosphorescence intensity of $(Ph_4P)_2Cd_2Cl_6$ when excited at 254 nm and 365 nm.

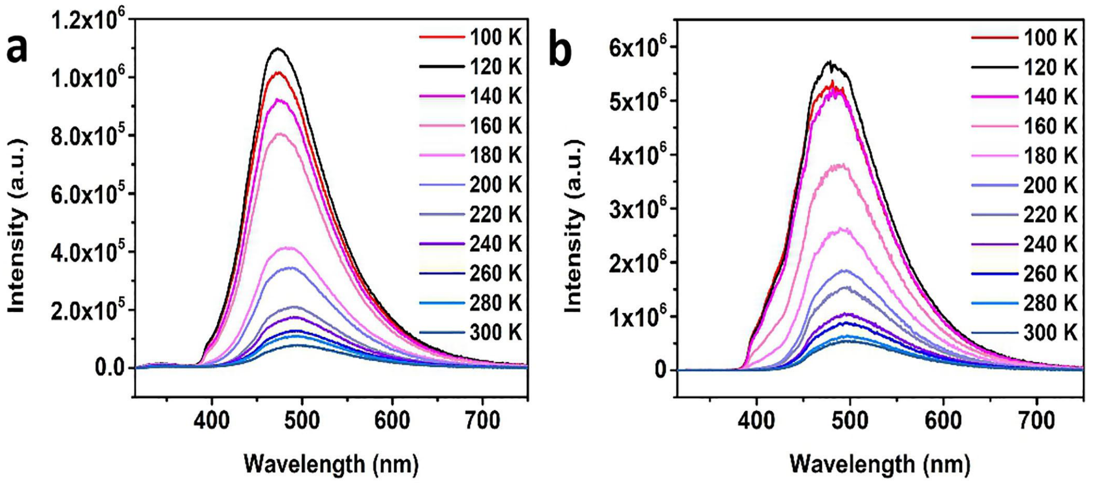

Supplementary Figure 8 | Solid-state PL spectra of Ph_4PBr (100 K–300 K) excited at 300 nm for (a) prompt and (b) delayed modes.

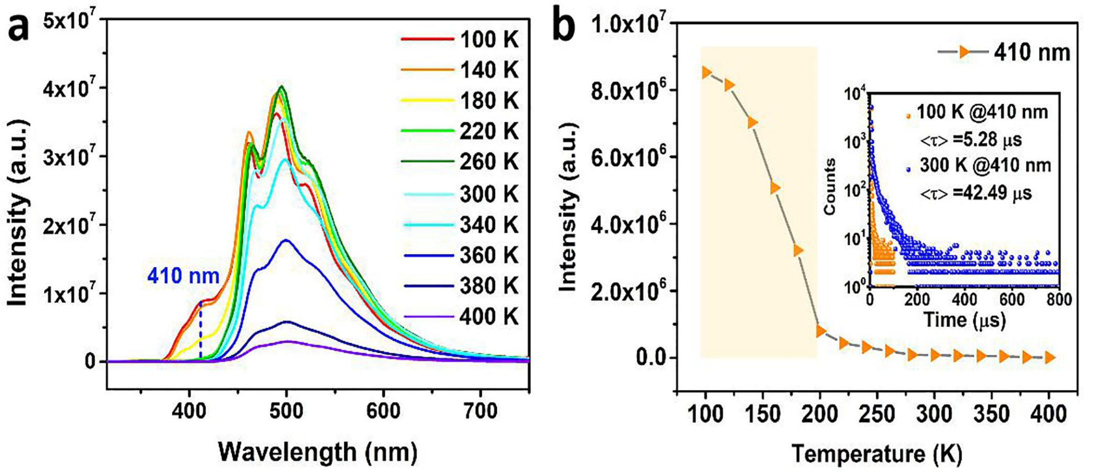

Supplementary Figure 9 | (a) Solid-state PL delayed spectra of $(Ph_4P)_2Cd_2Br_6$ at different temperatures ranging from 100 to 400 K (λ_{ex}=300 nm); (b) Temperature-dependent intensity at 410 nm of $(Ph_4P)_2Cd_2Br_6$ (λ_{ex}=300 nm), the inset is PL lifetime of 410 nm at 100 K and 300 K.

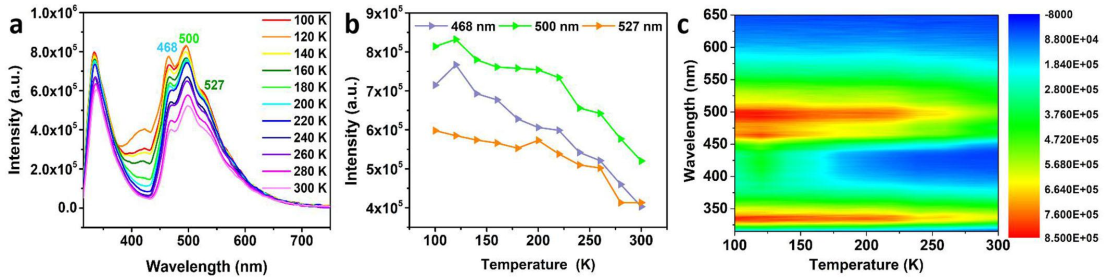

Supplementary Figure 10 | Photoemission properties of $(Ph_4P)_2Cd_2Cl_6$: (a) PL spectra; (b) Temperature-dependent intensity of emission (468 nm, 500 nm, and 527 nm) in the range from 100 to 300 K; (c) 2D emission contour spectra. (λ_{ex}=300 nm)

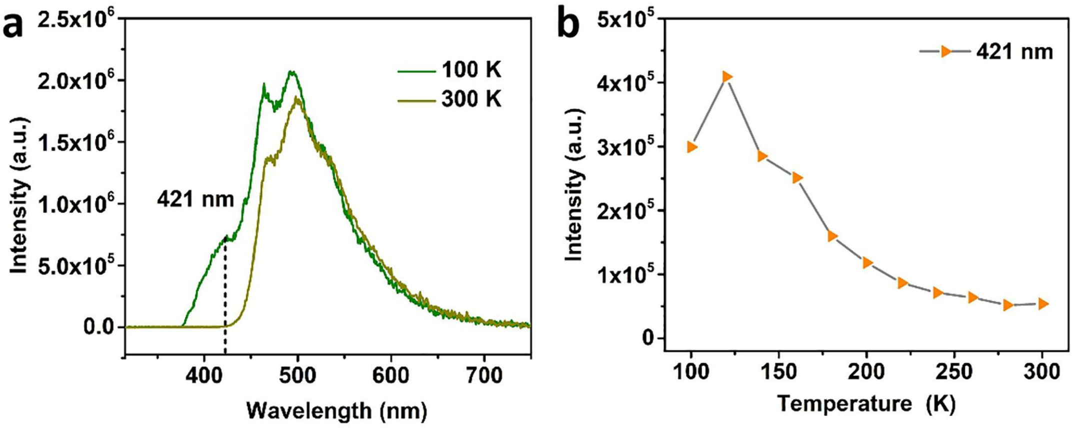

Supplementary Figure 11 | (a) Delayed PL spectra of $(Ph_4P)_2Cd_2Cl_6$; (b) Luminescence intensity at 421 nm in the temperature range 100–300 K. (λ_{ex}=300 nm)

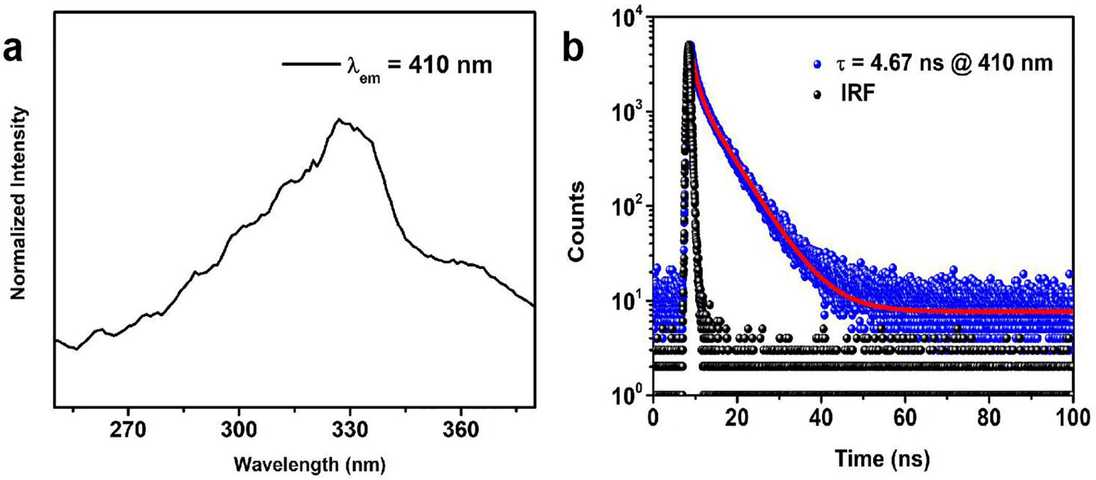

Supplementary Figure 12 | (a) Excited PL spectrum and (b) time-resolved PL decay curve of $(Ph_4P)_2Cd_2Br_6$ crystals at 410 nm.

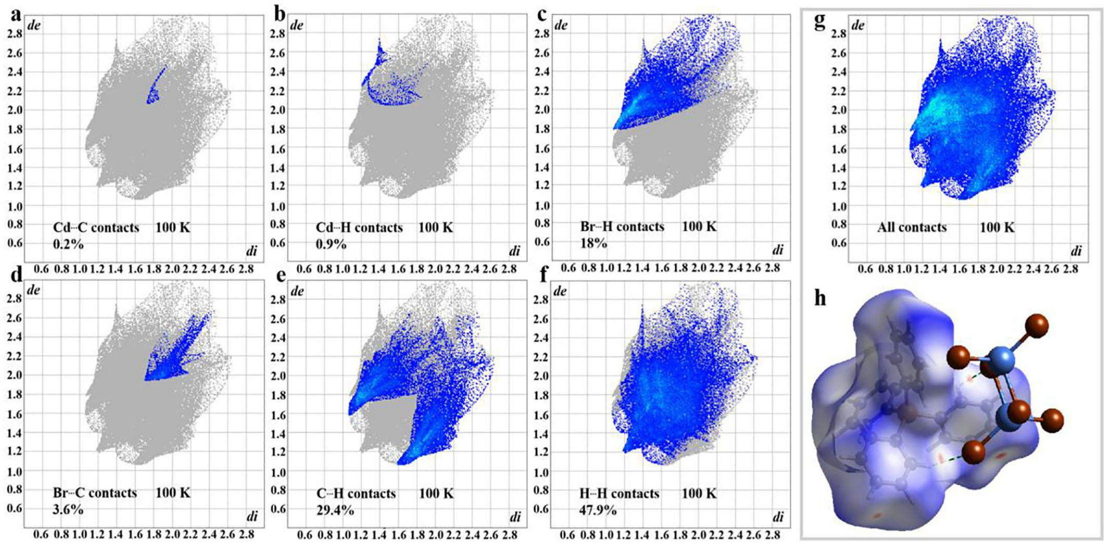

Supplementary Figure 13 | Two-dimensional fingerprint plot analysis: (a) Cd···C contacts, (b) Cd···H contacts, (c) Br···H contacts, (d) Br···C contacts, (e) C···H contacts, (f) H···H contacts, and (g) all contacts. (h) Hirshfeld surface mapped over d_{norm} highlighting the regions of Br···H hydrogen bonding (100 K).

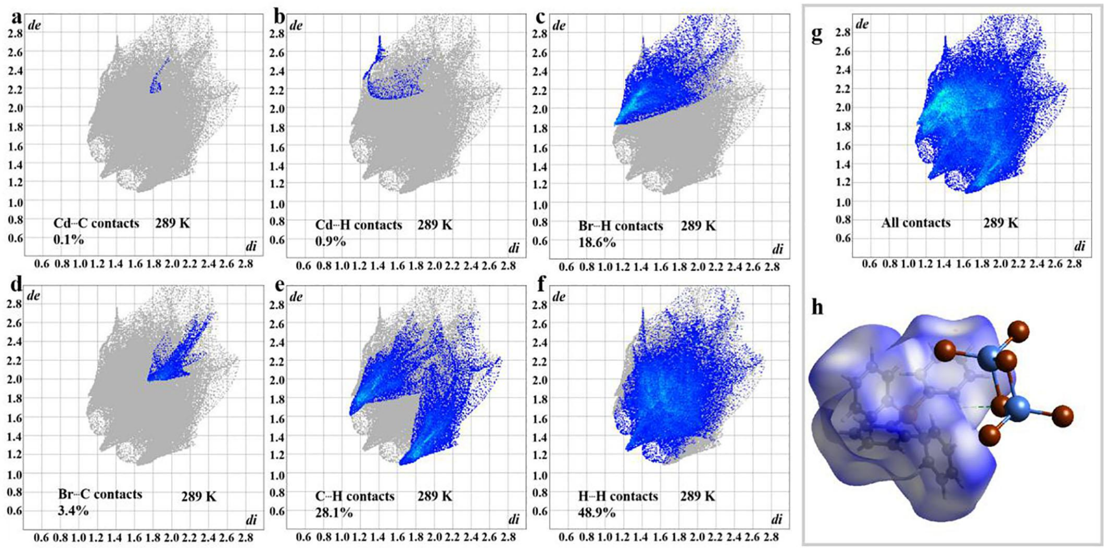

Supplementary Figure 14 | Two-dimensional fingerprint plot analysis: (a) Cd···C contacts, (b) Cd···H contacts, (c) Br···H contacts, (d) Br···C contacts, (e) C···H contacts, (f) H···H contacts, and (g) all contacts. (h) Hirshfeld surface mapped over d_{norm} highlighting the regions of Br···H hydrogen bonding (289 K).

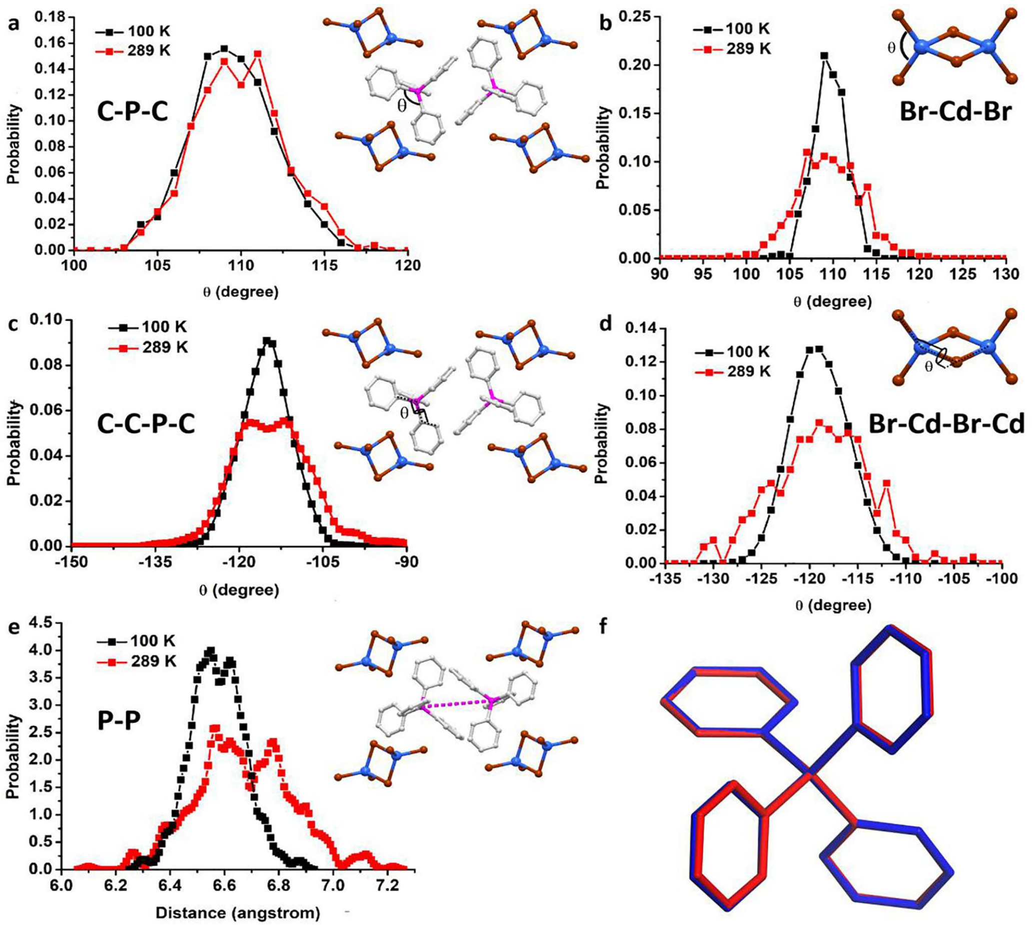

Supplementary Figure 15 | The distributions of orientational angle θ of $(Ph_4P)_2Cd_2Br_6$ at 100 K and 289 K: (a)∠CPC, (b)∠BrCdBr, (c) dihedral angle of C–C–P–C, (d) dihedral angle of Br-Cd-Br-Cd. (e) The distribution of the central distance between the two nearest neighboring Ph_4P^+ cations. (f) Super imposition of the two single crystal structures at different temperatures (100 K, red; 289 K, blue). The protons are removed for the sake of clarity.

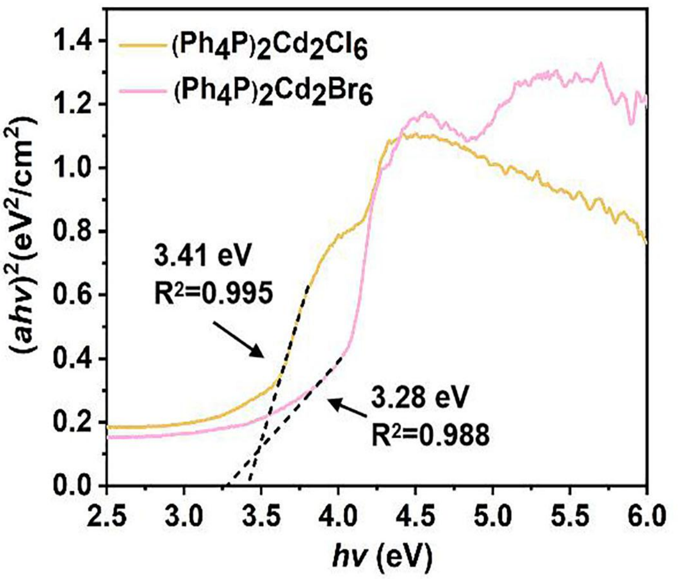

Supplementary Figure 16 | The experiment band gap result based on UV-vis absorption spectra.

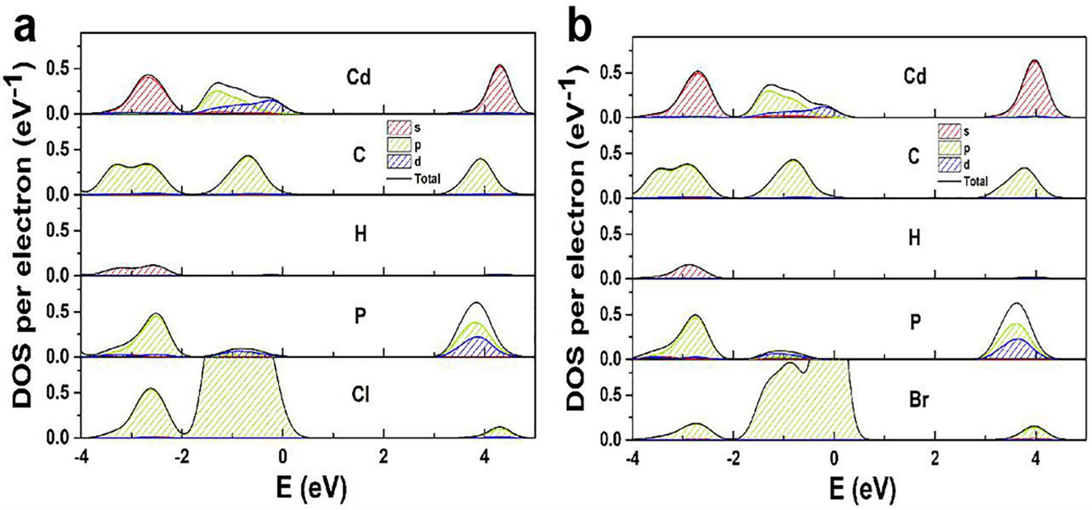

Supplementary Figure 17 | PDOS of different elements in $(Ph_4P)_2Cd_2Cl_6$ (a) and $(Ph_4P)_2Cd_2Br_6$ (b).

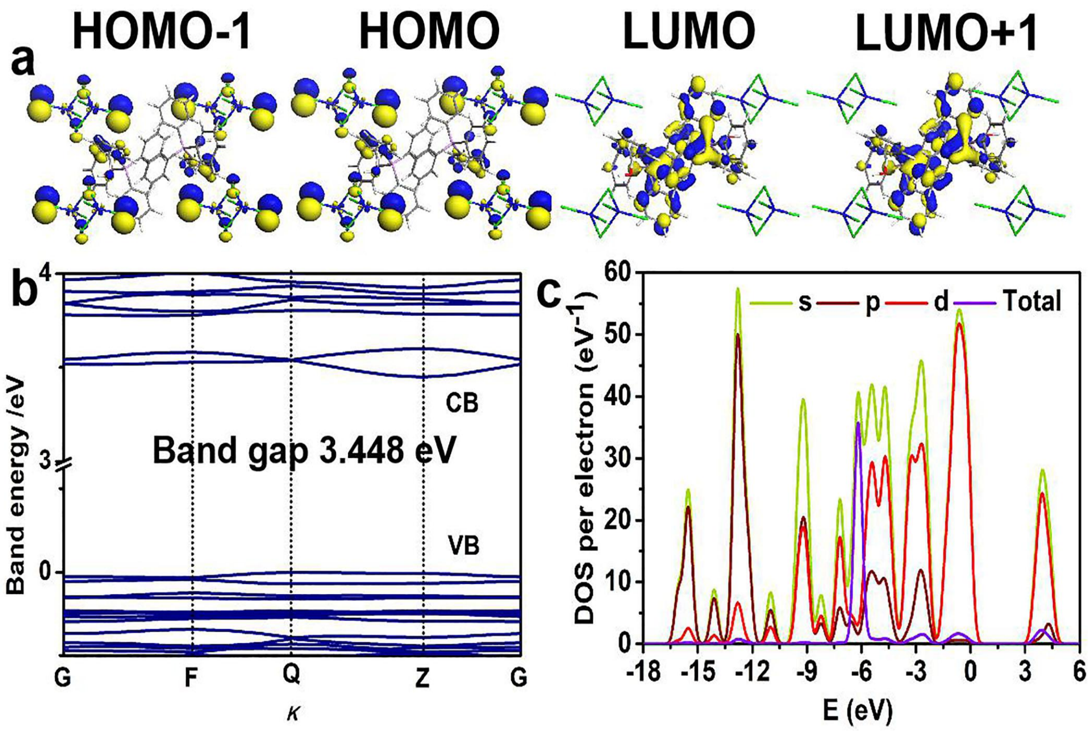

Supplementary Figure 18 | The calculated molecular orbitals (a), the band structures around Fermi energy level (b) and the total/partial electronic density of states (c) for $(Ph_4P)_2Cd_2Cl_6$,. G (0, 0, 0), F (0, 1/2, 0), Q (1/2, 0, 0), and Z (0, 0, 1/2) are the selected reciprocal points in the first Brillouin Zone (BZ).

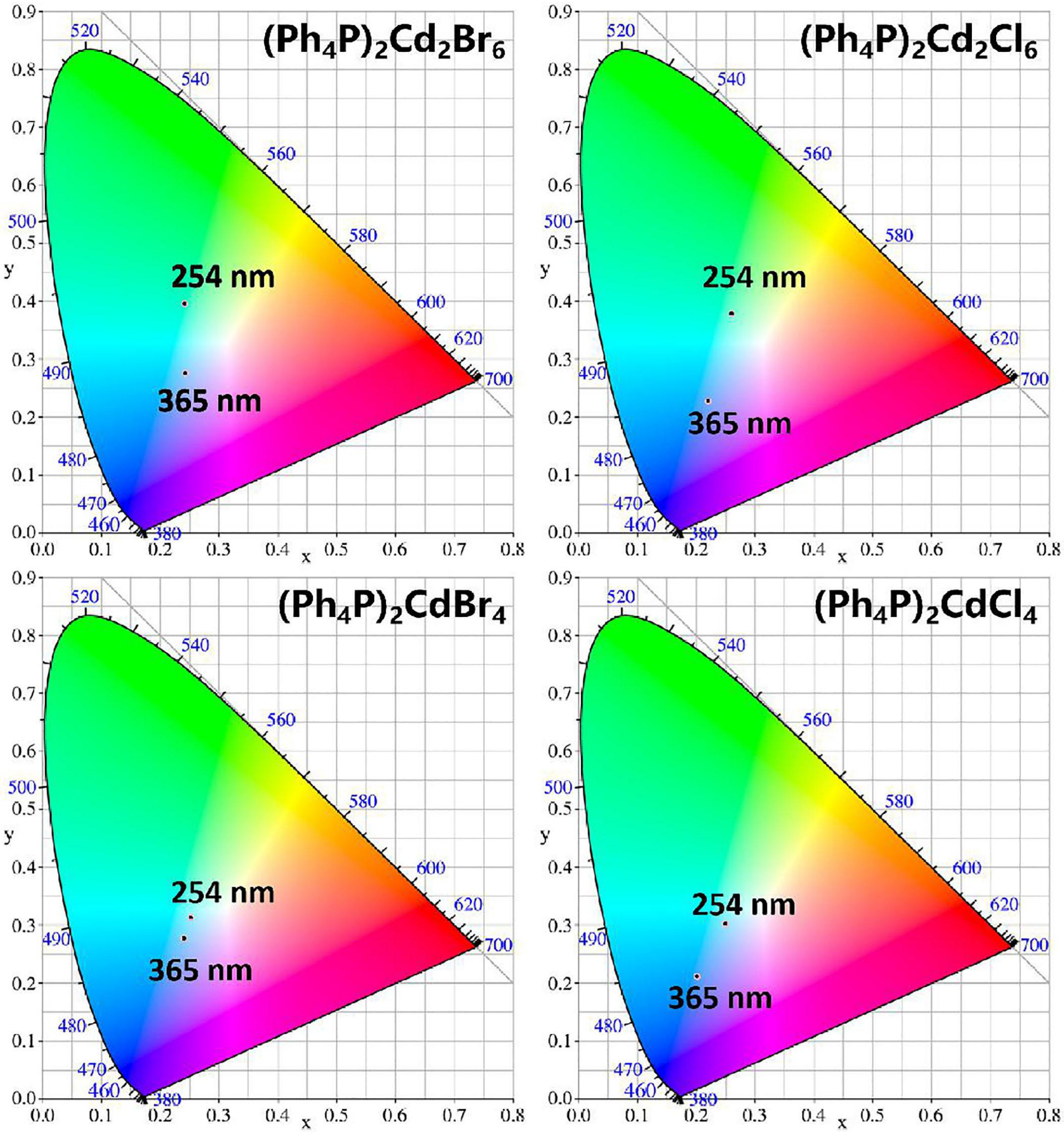

Supplementary Figure 19 | Luminescent color coordination of different samples under different excitation wavelengths.

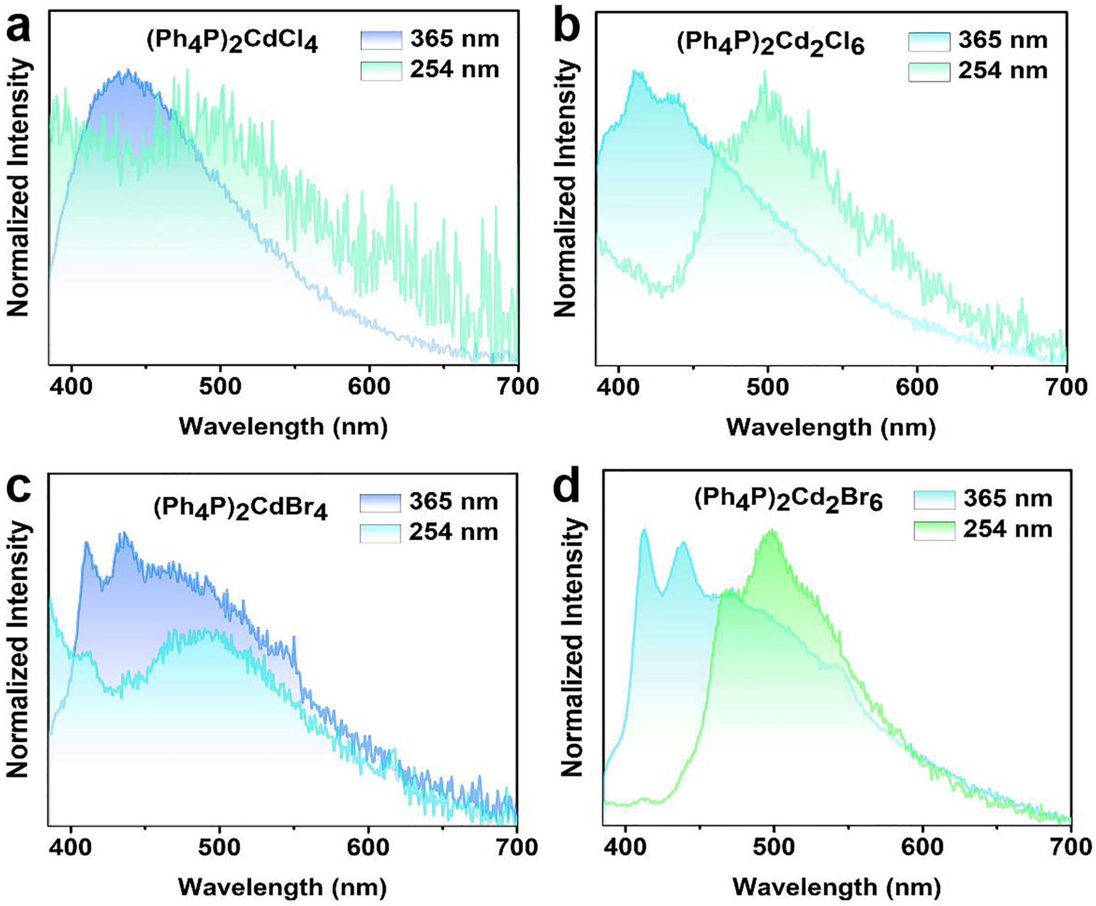

Supplementary Figure 20 | PL spectra of different samples under different excitation wavelengths.

Supplementary Table 1 | Examples of the variation of fluorescence or phosphorescence with increasing temperature.

Compound	Temperature range (K)	Variation tendency	λ_{ex} (nm)	λ_{em} (nm)	Emission intensity percentage at 300 K (approximate results)	Ref.
**1·NH_2CH_3**	30-298	↗↘	370	524, 641	40%	1.
**2·NH_2CH_3**	30-298	↗↘	370	Appr.550	40%	
**Zn_5 MOF**	77-420	↘	365	500	25%	2.
**CZ-DBFBr**	4-300	↘	400	500	0	3.
		↗↘		655	1.875	
**$CdCl_2$-4HP**	100-300	↘	365	480	40%	4.
**PA-ME**	220-340	↘	280	540	40%	5.
**IPA-ME**		↘		524	40%	
**TPA-ME**		↘		554	70%	
**NCD1-C**	305-433	↘	365	500	-	6.
**MCDF-1G**	77-427	↘	/	540	40%	7.
**BFPtPZ**	304-322	↗	360	550-750	/	8.
**1'-Eu**	90-300	↘	381	$^5D_0 \rightarrow {}^7F_2$	30%	9.
**1'-Tb**		↗	356	$^5D_4 \rightarrow {}^7F_5$	1.2	
**1'-$Eu_{0.015}Tb_{0.985}$**		↘	342	$^5D_0 \rightarrow {}^7F_2$	20%	
				$^5D_4 \rightarrow {}^7F_5$	50%	
**TbPIA**	14-300	↘	360	$^5D_4 \rightarrow {}^7F_5$	40%	10.

**EuPIA**		↘		$^5D_0 \to {}^7F_2$	80%	
**NPs**	283-323	↘	400	616	20%	11.
**ZJU-88⊃perylene**	293-353	↗	388	615	-	12.
**P1**	285-313	↗	405	590	2.9	13.
**P2**				470	8.6	
**P3**				470	10	
				590	1.8	
**Pd-O-S**	278-353	↘	465	772	25%	14.
**ClBDBT**	50-300	↘	-	467	50%	15.
				551	33.3%	
**m-CzPh**	173-293	↘	-	520	12.5%	16.
				550	65%	

Supplementary Table 2 | Crystal data and structure refinement for $(Ph_4P)_2CdX_4$.

**Samples**	**$(Ph_4P)_2CdCl_4$ (CCDC:1971490)**	**$(Ph_4P)_2CdBr_4$ (CCDC:1971489)**
**Formula**	$C_{96}H_{82}Cd_2Cl_8OP_4$	$C_{24}H_{20}Br_2Cd_{0.5}P$
Mr	1883.89	555.39
**Temperature (K)**	100.01(10)	100.00(10)
**Crystal system**	monoclinic	monoclinic
**Space group**	*C2/c*	*C2/c*
**Crystal size (mm)**	0.15 × 0.10 × 0.06	0.10 × 0.08 × 0.06
a **(Å)**	11.04210(10)	11.27250(10)
b **(Å)**	19.2314(2)	19.5714(3)
c **(Å)**	20.3090(2)	20.5184(3)
α **(°)**	90	90
β **(°)**	91.3590(10)	92.1270(10)
γ **(°)**	90	90
**V(Å^3)**	4311.51(7)	4523.62(10)
Z	2	8
**D_{calc} (mg/m^3)**	1.451	1.631
**θ Range (°)**	4.36-71.992	4.312-71.993
**F (000)**	1916.0	2184.0
**Data/restraint/parameters**	4208 / 1 / 257	4391 / 0 / 249
**Reflections collected**	14582	15303
**Independent reflections**	4208	4391
**Goodness-of-fit on F^2**	1.036	1.045
**R_{int}**	0.0190	0.0407
**$R_1[I>2\sigma(I)]$**	0.0231	0.0266
**$wR_2[I>2\sigma(I)]$**	0.0625	0.0687
**R_1(all data)**	0.0233	0.0274
**wR_2(all data)**	0.0626	0.0692
**Residuals(e Å^{-3})**	0.67, -0.67	1.10, -0.69

$R_1 = \Sigma||F_o| - |F_c||/\Sigma|F_o|$, $wR_2 = [\Sigma_w(F_o^2 - F_c^2)^2/\Sigma w(F_o^2)^2]^{1/2}$

Supplementary Table 3 | Crystal data and structure refinement for $(Ph_4P)_2Cd_2X_6$.

**Samples**	**$(Ph_4P)_2Cd_2Cl_6$ (CCDC:1971488)**	**$(Ph_4P)_2Cd_2Br_6$ (CCDC:1971491)**
**Formula**	$C_{24}H_{20}PO_{0.5}CdCl_3$	$C_{24}H_{20}PCdBr_3$
Mr	567.13	691.50
**Temperature (K)**	100.01(10)	100.01(10)
**Crystal system**	triclinic	triclinic
**Space group**	$P\bar{1}$	$P\bar{1}$
**Crystal size (mm)**	0.17 × 0.11 × 0.05	0.30 × 0.20 × 0.06
a **(Å)**	9.6719(3)	9.8553(3)
b **(Å)**	9.8344(5)	9.9994(3)
c **(Å)**	12.9549(6)	13.0671(4)
α **(°)**	108.624(4)	108.652(2)
β **(°)**	93.881(3)	93.996(2)
γ **(°)**	100.033(3)	100.039(6)
**V(Å^3)**	1139.77(9)	1190.50(6)
Z	2	2
**Dcalc (mg/m^3)**	1.653	1.929
**θ Range (°)**	3.632-72.074	3.603-71.69
**F (000)**	566	664.0
**Data/restraint/parameters**	4344 / 0 / 274	4533 / 0 / 262
**Reflections collected**	7826	7940
**Independent reflections**	4344	4533
**Goodness-of-fit on F^2**	1.092	1.072
**R_{int}**	0.0424	0.0472
**$R_1[I>2\sigma(I)]$**	0.0465	0.0436
**wR_2[I>2σ(I)]**	0.1148	0.1144
**R_1(all data)**	0.0472	0.0444
**wR_2(all data)**	0.1153	0.1155
**Residuals(e Å^{-3})**	2.15, -2.88	1.91, -1.81

$R_1 = \Sigma||F_o| - |F_c||/\Sigma|F_o|$, $wR_2 = [\Sigma_w(F_o^2 - F_c^2)^2/\Sigma w(F_o^2)^2]^{1/2}$

Supplementary Table 4 | Distance between Ph_4P^+ cations and quantum yield of Ph_4PCl and Ph_4PBr.

Samples	Distance between Ph-Ph (Å)	Distance between Ph_4P^+ cations (Å)	RTP Quantum yield
**Ph_4PCl**	3.04	7.72	8.02%
**Ph_4PBr**	3.33	8.18	21.77%

Supplementary Table 5 | Distance between Ph_4P^+ cations and distance between dimers.

Samples	Distance (Å)	Dimer(near) (Å)	Dimer (far) (Å)
**$(Ph_4P)_2Cd_2Cl_6$**	6.468	9.834	12.955
**$(Ph_4P)_2Cd_2Br_6$**	6.585	9.999	13.067
**$(Ph_4P)_2CdCl_4$**	6.238	7.159	11.088
**$(Ph_4P)_2CdBr_4$**	6.357	7.287	11.293
**$(Ph_4P)_2Pb_3Cl_8$**	8.161	11.698	15.104

Supplementary Table 6 | The comparison of the long-lived lifetime and quantum yield values in refs.[17-35] and this work under ambient conditions.

Compound	Lifetime	Quantum Yield	Ref.
**PEPB-NIA**	6.3 ms	25.6%	17.
**PENC-NIA**	35 ms	56.1%	
**PTPB**	3.04 ms	11.2%	18.
**PNA**	173 ms	17.0%	19.
**Br-PNA**	64.8 ms	14.0%	
**PNA-Br**	7.3 ms	22.0%	
**PYCl/CB[6]**	5400 μs	81.2%	20.
**PYBr/CB[6]**	8748 μs	72.9%	
**PYI/CB[6]**	7135 μs	3.0%	
**PSS2000000-100%**	1221 ms	3.6%	21.
**1,4-benzenediboronic acid bis(pinacol)ester (KBr)**	1.85 s	2.0%	22.
**H-NpCzBF$_2$ (powder)**	0.13 ms	36.2%	23.
**Br-NpCzBF$_2$ (powder)**	0.58 ms	25.9%	
**I-NpCzBF$_2$ (powder)**	0.56 ms	7.4%	
**H-NpCzBF$_2$ (nano particles)**	29.0 μs	23.1%	
**Br-NpCzBF$_2$ (nano particles)**	27.6 μs	6.5%	
**I-NpCzBF$_2$ (nano particles)**	17.2 μs	3.6%	
**9-(4-(phenylsulfonyl)phenyl)- 9H-carbazole (0M)**	0.36 s	1.50%	24.
**1M**	0.82 s	2.64%	
**9-(4-(mesitylsulfonyl)phenyl)- 9H-carbazole (3M)**	0.83 s	1.11%	
**9-(dibenzo[b,d]furan-2-yl)- 9H-carbazole**	0.65 s	14.3%	25.
**9-(8-bromodibenzo[b,d]furan2-yl)- 9H-carbazole**	0.54 s	41.2%	
**9-(dibenzo[b,d]thiophen-2- yl)-9H-carbazole**	0.45 s	10.1%	
**9-(8- bromodibenzo[b,d]thiophen2-yl)-9H-carbazole**	0.42 s	12.1%	
**1-(dibenzo[b,d]furan-2-yl) phenylmethanone**	232 ms	34.5%	26.
**10-phenyl-10Hphenothiazine 5,5-dioxide derivatives (CSCl)**	256 ms	10.16%	27.
**1-(5, 5-dioxido-10Hphenothiazin-10-yl)ethan-1- one**	876 ms	8.2%	28.
**PhTCz-1**	79.83 ms	4.0%	29.
**4,6-diphenyl-2-carbazolyl1,3,5-triazine**	1.06 s	1.25%	30.
**2,4,6-trimethoxy-1,3,5- triazine**	0.58 s/0.75 s	31.2%	31.
**2-chloro-4,6-dimethoxy1,3,5-triazine**	2.45 s	7.2 %	
**MA-IPA**	1.91 s	24.3%	32.

**PBC/CB[6] complex**	2.62 s	9.7%	33.
**Isophthalate/LDHs**	1.23 s	3.02%	34.
**1-DMF**	472 ms	4.76%	35.
**MOF-5**	153 ms	0.22%	
**$(Ph_4P)_2Cd_2Br_6$**	37.85 ms	62.79%	This work

Supplementary Table 7 | Transient PL time constants and the pre-exponential coefficients.

**Temp**	**Sample**	λ_{ex} **(nm)**	λ_{em} **(nm)**	τ_i	A_i **(%)**	$\langle\tau\rangle$	χ^2
**RT**	Ph_4PCl	300	500	262.1 ms	26.91	0.66 s	1.196
				809.4 ms	73.09		
**RT**	Ph_4PBr	300	500	286.4 μs	66.89	1.51 ms	1.291
				3995 μs	33.02		
**RT**	$(Ph_4P)_2Cd_2Cl_6$	300	500	210.3 ms	11.58	0.82 s	1.182
				900.3 ms	88.42		
**RT**	$(Ph_4P)_2Cd_2Br_6$	300	500	37.850 ms	100	37.85 ms	1.222
**100 K**			410	5.28 μs	100	5.28 μs	1.280
**300 K**			410	42.49 μs	100	42.49 μs	0.943
**100 K**			500	17.58 ms	43.48	49.58 ms	1.258
				74.15 ms	56.52		
**200 K**				16.07 ms	31.88	39.19 ms	1.267
				50.00 ms	68.12		
**300 K**				9.27 ms	13.72	33.50 ms	1.238
				37.35 ms	86.28		
**400 K**				3.67 ms	100	3.67 ms	1.027
**RT**		332.2	410	1.19 ns	27.53	4.67 ns	1.291
				6.00 ns	72.47		

τ_i is the excited state lifetime. A_i represents the ratio of τ_i. The fitting goodness is manifested by the value of χ^2. In the double-exponential case, $\langle\tau\rangle = A_1\tau_1 + A_2\tau_2$, $A_1 + A_2 = 1$. In the three-exponential case, $\langle\tau\rangle = A_1\tau_1 + A_2\tau_2 + A_3\tau_3$, $A_1 + A_2 + A_3 = 1$.

Supplementary Table 8 | Crystal data and structure refinement for $(Ph_4P)_2Cd_2Br_6$.

**Samples**	**$(Ph_4P)_2Cd_2Br_6$ 100 K （CCDC:1971491）**	**$(Ph_4P)_2Cd_2Br_6$ 289 K (CCDC:1971487)**
**Formula**	$C_{24}H_{20}PCdBr_3$	$C_{24}H_{20}PCdBr_3$
Mr	691.50	691.50
**Temperature (K)**	100.01(10)	289(1)
**Crystal system**	triclinic	triclinic
**Space group**	$P\bar{1}$	$P\bar{1}$
**Crystal size (mm)**	0.30 × 0.20 × 0.06	0.1 × 0.07 × 0.05
a **(Å)**	9.8553(3)	9.9955(3)
b **(Å)**	9.9994(3)	10.1202(3)
c **(Å)**	13.0671(4)	13.1297(4)
α **(°)**	108.652(2)	108.556(3)
β **(°)**	93.996(2)	94.149(3)
γ **(°)**	100.039(6)	100.371(3)
**V(Å^3)**	1190.50(6)	1227.51(7)
**Z**	2	2
**D_{calc} (mg/m^3)**	1.929	1.872
**θ Range (°)**	3.603-71.69	3.586-72.003
**F (000)**	664.0	664.0
**Data/restraint/parameters**	4533 / 0 / 262	4659 / 0 / 262
**Reflections collected**	7940	8294
**Independent reflections**	4533	4659
**Goodness-of-fit on F^2**	1.072	1.116
**R_{int}**	0.0472	0.0231
**R_1[I>2σ(I)]**	0.0436	0.0334
**wR_2[I>2σ(I)]**	0.1144	0.0798
**R_1(all data)**	0.0444	0.0377
**wR_2(all data)**	0.1155	0.0817
**Residuals(e Å^{-3})**	1.91, -1.81	0.47, -1.26

$R_1 = \Sigma||F_o| - |F_c||/\Sigma|F_o|$, $wR_2 = [\Sigma_w(F_o^2 - F_c^2)^2/\Sigma w(F_o^2)^2]^{1/2}$

Supplementary Table 9 | Selected bond lengths (Å) and angles (o) for $(Ph_4P)_2Cd_2Br_6$ (100 K).

Bond/angle	lengths (Å)/angle (o)	Bond/angle (o)	lengths (Å)
**Cd(1)-Br(2)**	2.5253(5)	**C(14)-C(15)**	1.396(6)
**Cd(1)-Br(3)**	2.5421(5)	**C(13)-C(18)**	1.390(6)
**Cd(1)-Br(1)**	2.6653(5)	**C(5)-C(6)**	1.391(6)
**Cd(1)-Br(1)#1**	2.6789(5)	**C(5)-C(4)**	1.397(6)
**Br(1)-Cd(1)#1**	2.6790(5)	**C(3)-C(4)**	1.390(7)
**P(1)-C(19)**	1.790(4)	**C(3)-C(2)**	1.390(6)
**P(1)-C(13)**	1.796(4)	**C(2)-C(1)**	1.390(6)
**P(1)-C(1)**	1.799(4)	**C(19)-C(24)**	1.395(6)
**P(1)-C(7)**	1.800(4)	**C(10)-C(9)**	1.393(7)
**C(20)-C(19)**	1.381(6)	**C(10)-C(11)**	1.393(7)
**C(20)-C(21)**	1.396(6)	**C(22)-C(23)**	1.380(7)
**C(12)-C(11)**	1.385(6)	**C(1)-C(6)**	1.410(6)
**C(12)-C(7)**	1.396(6)	**C(16)-C(17)**	1.377(7)
**C(21)-C(22)**	1.397(6)	**C(16)-C(15)**	1.394(7)
**C(8)-C(9)**	1.393(6)	**C(17)-C(18)**	1.393(6)
**C(8)-C(7)**	1.396(6)	**C(24)-C(23)**	1.395(6)
**C(14)-C(13)**	1.389(6)		
**Br(2)-Cd(1)-Br(3)**	118.748(17)	**C(1)-C(2)-C(3)**	119.6(4)
**Br(2)-Cd(1)-Br(1)**	111.142(16)	**C(20)-C(19)-C(24)**	120.4(4)
**Br(3)-Cd(1)-Br(1)**	111.492(17)	**C(20)-C(19)-P(1)**	121.5(3)
**Br(2)-Cd(1)-Br(1)#1**	112.328(16)	**C(24)-C(19)-P(1)**	118.0(3)
**Br(3)-Cd(1)-Br(1)#1**	107.719(17)	**C(9)-C(10)-C(11)**	120.2(4)
**Br(1)-Cd(1)-Br(1)#1**	92.239(14)	**C(23)-C(22)-C(21)**	120.5(4)
**Cd(1)-Br(1)-Cd(1)#1**	87.761(14)	**C(2)-C(1)-C(6)**	120.0(4)
**C(19)-P(1)-C(13)**	111.83(19)	**C(2)-C(1)-P(1)**	122.9(3)
**C(19)-P(1)-C(1)**	110.18(19)	**C(6)-C(1)-P(1)**	117.0(3)
**C(13)-P(1)-C(1)**	106.92(18)	**C(3)-C(4)-C(5)**	119.6(4)
**C(19)-P(1)-C(7)**	106.56(19)	**C(17)-C(16)-C(15)**	120.0(4)
**C(13)-P(1)-C(7)**	109.25(19)	**C(12)-C(7)-C(8)**	120.4(4)
**C(1)-P(1)-C(7)**	112.16(19)	**C(12)-C(7)-P(1)**	122.2(3)
**C(19)-C(20)-C(21)**	120.0(4)	**C(8)-C(7)-P(1)**	117.1(3)
**C(11)-C(12)-C(7)**	120.0(4)	**C(16)-C(17)-C(18)**	119.8(4)
**C(22)-C(21)-C(20)**	119.4(4)	**C(12)-C(11)-C(10)**	119.8(4)
**C(9)-C(8)-C(7)**	119.2(4)	**C(23)-C(24)-C(19)**	119.6(4)

**C(13)-C(14)-C(15)**	118.6(4)	**C(16)-C(15)-C(14)**	121.0(4)
**C(14)-C(13)-C(18)**	121.2(4)	**C(17)-C(18)-C(13)**	119.4(4)
**C(14)-C(13)-P(1)**	122.4(3)	**C(10)-C(9)-C(8)**	120.3(4)
**C(18)-C(13)-P(1)**	116.4(3)	**C(22)-C(23)-C(24)**	120.0(4)
**C(6)-C(5)-C(4)**	120.1(4)	**C(5)-C(6)-C(1)**	119.7(4)
**C(4)-C(3)-C(2)**	120.9(4)		

Symmetry codes: #1 -x+2,-y+1,-z.

Supplementary Table 10 | Selected bond lengths (Å) and angles (°) for $(Ph_4P)_2Cd_2Br_6$ (289 K).

Bond/angle	lengths (Å)/angle (o)	Bond/angle (°)	lengths (Å)
**Cd(1)-Br(2)**	2.5214(5)	**C(14)-C(15)**	1.395(6)
**Cd(1)-Br(3)**	2.5331(5)	**C(13)-C(18)**	1.393(5)
**Cd(1)-Br(1)**	2.6686(5)	**C(5)-C(6)**	1.372(6)
**Cd(1)-Br(1)#1**	2.6823(5)	**C(5)-C(4)**	1.383(7)
**Br(1)-Cd(1)#1**	2.6824(5)	**C(3)-C(4)**	1.372(7)
**P(1)-C(19)**	1.793(3)	**C(3)-C(2)**	1.399(6)
**P(1)-C(13)**	1.794(4)	**C(2)-C(1)**	1.382(5)
**P(1)-C(1)**	1.796(3)	**C(19)-C(24)**	1.391(5)
**P(1)-C(7)**	1.801(3)	**C(10)-C(9)**	1.374(8)
**C(20)-C(19)**	1.384(5)	**C(10)-C(11)**	1.380(7)
**C(20)-C(21)**	1.386(5)	**C(22)-C(23)**	1.370(7)
**C(12)-C(11)**	1.377(6)	**C(1)-C(6)**	1.394(5)
**C(12)-C(7)**	1.386(6)	**C(16)-C(17)**	1.369(8)
**C(21)-C(22)**	1.371(6)	**C(16)-C(15)**	1.374(8)
**C(8)-C(9)**	1.383(7)	**C(17)-C(18)**	1.382(6)
**C(8)-C(7)**	1.388(5)	**C(24)-C(23)**	1.384(6)
**C(14)-C(13)**	1.378(5)		
**Br(2)-Cd(1)-Br(3)**	118.361(19)	**C(1)-C(2)-C(3)**	118.8(4)
**Br(2)-Cd(1)-Br(1)**	111.502(17)	**C(20)-C(19)-C(24)**	120.1(3)
**Br(3)-Cd(1)-Br(1)**	111.508(18)	**C(20)-C(19)-P(1)**	121.7(3)
**Br(2)-Cd(1)-Br(1)#1**	111.734(16)	**C(24)-C(19)-P(1)**	118.1(3)
**Br(3)-Cd(1)-Br(1)#1**	108.577(17)	**C(9)-C(10)-C(11)**	120.3(4)
**Br(1)-Cd(1)-Br(1)#1**	92.046(13)	**C(23)-C(22)-C(21)**	120.1(4)
**Cd(1)-Br(1)-Cd(1)#1**	87.955(13)	**C(2)-C(1)-C(6)**	120.1(3)
**C(19)-P(1)-C(13)**	109.56(15)	**C(2)-C(1)-P(1)**	122.0(3)
**C(19)-P(1)-C(1)**	111.88(16)	**C(6)-C(1)-P(1)**	117.8(3)
**C(13)-P(1)-C(1)**	106.34(16)	**C(3)-C(4)-C(5)**	120.3(4)
**C(19)-P(1)-C(7)**	106.76(16)	**C(17)-C(16)-C(15)**	120.1(4)
**C(13)-P(1)-C(7)**	112.21(16)	**C(12)-C(7)-C(8)**	119.4(4)
**C(1)-P(1)-C(7)**	110.16(16)	**C(12)-C(7)-P(1)**	117.5(3)
**C(19)-C(20)-C(21)**	119.4(3)	**C(8)-C(7)-P(1)**	122.9(3)
**C(11)-C(12)-C(7)**	120.7(4)	**C(16)-C(17)-C(18)**	120.0(4)
**C(22)-C(21)-C(20)**	120.5(4)	**C(12)-C(11)-C(10)**	119.5(4)
**C(9)-C(8)-C(7)**	119.6(4)	**C(23)-C(24)-C(19)**	119.3(4)
**C(13)-C(14)-C(15)**	118.8(4)	**C(16)-C(15)-C(14)**	120.9(4)

**C(14)-C(13)-C(18)**	120.2(4)	**C(17)-C(18)-C(13)**	120.0(4)
**C(14)-C(13)-P(1)**	122.7(3)	**C(10)-C(9)-C(8)**	120.4(4)
**C(18)-C(13)-P(1)**	117.1(3)	**C(22)-C(23)-C(24)**	120.6(4)
**C(6)-C(5)-C(4)**	119.8(4)	**C(5)-C(6)-C(1)**	120.4(4)
**C(4)-C(3)-C(2)**	120.6(4)		

Symmetry codes: #1 -x+2,-y+1,-z.

Supplementary Table 11 | Selected distance measured in the $(Ph_4P)_2Cd_2Br_6$ between 100 and 289 K.

Bonds	Distance (Å) 100 K	Distance (Å) 289 K	Change (Å)
**Cd-Cd(in the cluster)**	3.7045	3.7156	0.0111
**Cd-Cd(between the cluster)**	9.8553	9.9955	0.1402
**P-P(in the dimer)**	6.5843	6.6360	0.0517
**P-P(between the dimer)**	9.9994	10.1202	0.1208

Supplementary Table 12 | Connolly surface results of $(Ph_4P)_2Cd_2Br_6$ under different temperatures.

Temperature	V_f (Å^3)	V_o (Å^3)	FFV (%)
**100 K**	93.13	1097.37	7.82
**289 K**	114.03	1112.48	9.30

Supplementary References

1. Zhan, S. Z., Li, M., Ng, S. W., & Li, D. Luminescent metal-organic frameworks (MOFs) as a chemopalette: tuning the thermochromic behavior of dual-emissive phosphorescence by adjusting the supramolecular microenvironments. *Chem. Eur. J.* **19,** 10217-10225 (2013).

2. Li, D., Yang, X., & Yan, D. Cluster-based metal-organic frameworks: modulated singlet-triplet excited states and temperature-responsive phosphorescent switch. *ACS Appl. Mater. Interfaces.* **10,** 34377-34384 (2018).

3. Zhao, W. et al. Boosting the efficiency of organic persistent room-temperature phosphorescence by intramolecular triplet-triplet energy transfer. *Nat. Commun.* **10,** 1595 (2019).

4. Zhou, B., & Yan, D. Simultaneous long–persistent blue luminescence and high quantum yield within 2D organic–metal halide perovskite micro/nanosheets. *Angew. Chem. Int. Ed.* **131,** 2-10 (2019).

5. Zhou, B., & Yan, D. Room-temperature phosphorescence: hydrogen-bonded two-component ionic crystals showing enhanced long-lived room-temperature phosphorescence via TADF-assisted Förster resonance energy transfer. *Adv. Funct. Mater.* **29,** 1807599 (2019).

6. Lin, C., Zhuang, Y., Li, W., Zhou, T. L., & Xie, R. J. Blue, green, and red full-color ultralong afterglow in nitrogen-doped carbon dots. *Nanoscale.* **11,** 6584-6590 (2019).

7. Yang, Y., Yang, X., Fang, X., Wang, K. Z., & Yan, D. Reversible mechanochromic delayed fluorescence in 2D metal-organic micro/nanosheets: switching singlet-triplet states through transformation between exciplex and excimer. *Adv. Sci.* **5,** 1801187 (2018).

8. Han, M., Tian, Y., Yuan, Z., Zhu, L., & Ma, B. A phosphorescent molecular "butterfly" that undergoes a photoinduced structural change allowing temperature sensing and white emission. *Angew. Chem. Int. Ed.* **53,** 10908-10912 (2014).

9. Yao, J., Zhao, Y. W., & Zhang, X. M. Breathing europium-terbium co-doped luminescent MOF as a broad-range ratiometric thermometer with a contrasting temperature-intensity relationship. *ACS Omega.* **3,** 5754-5760 (2018).

10. Rao, X. et al. A highly sensitive mixed lanthanide metal-organic framework self-calibrated luminescent thermometer. *J. Am. Chem. Soc.* **135,** 15559-15564 (2013).

11. Peng, H., Stich, M. I. J., Yu, J., Sun, L., Fischer, L. H., & Wolfbeis, O. S. Luminescent europium (III) nanoparticles for sensing and imaging of temperature in the physiological range. *Adv. Mater.* **22,** 716-719 (2010).

12. Cui, Y. et al. Dual-emitting MOF ⊃ dye composite for ratiometric temperature sensing. *Adv. Mater.* **27,** 1420-1425 (2015).

13. Chen, Z. et al. Phosphorescent polymeric thermometers for in vitro and in vivo

temperature sensing with minimized background interference. *Adv. Funct. Mater.* **26,** 4386-4396 (2016).

14. Zach, P. W., Freunberger, S. A., Klimant, I., & Borisov, S. M. Electron-deficient near-infrared Pt (II) and Pd (II) benzoporphyrins with dual phosphorescence and unusually efficient thermally activated delayed fluorescence: First demonstration of simultaneous oxygen and temperature sensing with a single emitter. *ACS Appl. Mater. Interfaces.* **9,** 38008-38023 (2017).

15. He, Z. et al. White light emission from a single organic molecule with dual phosphorescence at room temperature. *Nat. Commun.* **8,** 416 (2017).

16. Ling, K. et al. Controllable multiemission with ultralong organic phosphorescence in crystal by isomerization. *Adv. Opt. Mater.* *7,* 1901076 (2019).

17. Yang, S. et al. Highly efficient room-temperature phosphorescence and afterglow luminescence from common organic fluorophores in 2D hybrid perovskites. *Chem. Sci.* **9,** 8975-8981 (2018).

18. Hu, H. et al. Efficient room-temperature phosphorescence from organic-inorganic hybrid perovskites by molecular engineering. *Adv. Mater.* **30,** 1707621 (2018).

19. Wei, J. et al. Induction of strong long-lived room-temperature phosphorescence of N-phenyl-2-naphthylamine molecules by confinement in a crystalline dibromobiphenyl matrix. *Angew. Chem. Int. Ed.* **55,** 15589-15593 (2016).

20. Zhang, Z. Y., Chen, Y., & Liu, Y. Efficient room-temperature phosphorescence of a solid-state supramolecule enhanced by cucurbit [6] uril. *Angew. Chem. Int. Ed.* **131,** 6089-6093 (2019).

21. Ogoshi, T. et al. Ultralong room-temperature phosphorescence from amorphous polymer poly (styrene sulfonic acid) in air in the dry solid state. *Adv. Funct. Mater.* **28,** 1707369 (2018).

22. Shoji, Y. et al. Unveiling a new aspect of simple arylboronic esters: long-lived room-temperature phosphorescence from heavy-atom-free molecules. *J. Am. Chem. Soc.* **139,** 2728-2733 (2017).

23. Wang, X. F. et al. Pure organic room temperature phosphorescence from excited dimers in self-assembled nanoparticles under visible and near-infrared irradiation in water. *J. Am. Chem. Soc.* **141,** 5045-5050 (2019).

24. Mao, Z. et al. The methylation effect in prolonging the pure organic room temperature phosphorescence lifetime. *Chem. Sci.* **10,** 179-184 (2019).

25. Zhao, W. et al. Boosting the efficiency of organic persistent room-temperature phosphorescence by intramolecular triplet-triplet energy transfer. *Nat. Commun.* **10,** 1595 (2019).

26. Zhao, W. et al. Rational molecular design for achieving persistent and efficient pure organic room-temperature phosphorescence. *Chem* **1,** 592-602 (2016).

27. Yang, J. et al. The influence of the molecular packing on the room temperature

phosphorescence of purely organic luminogens. *Nat. Commun.* **9,** 840 (2018).

28. Tian, S. et al. Utilizing d-pπ bonds for ultralong organic phosphorescence. *Angew. Chem. Int. Ed.* **58,** 6645-6649 (2019).

29. Cai, S. et al. Hydrogen-bonded organic aromatic frameworks for ultralong phosphorescence by intralayer π-π interactions. *Angew. Chem. Int. Ed.* **130,** 4069-4073 (2018).

30. An, Z. et al. Stabilizing triplet excited states for ultralong organic phosphorescence. *Nat. Mater.* **14,** 685-690 (2015).

31. Gu, L. et al. Colour-tunable ultra-long organic phosphorescence of a single-component molecular crystal. *Nat. Photonics.* **13,** 406-411 (2019).

32. Bian, L. et al. Simultaneously enhancing efficiency and lifetime of ultralong organic phosphorescence materials by molecular self-assembly. *J. Am. Chem. Soc.* **140,** 10734-10739 (2018).

33. Zhang, Z. Y., & Liu, Y. Ultralong room-temperature phosphorescence of a solid-state supramolecule between phenylmethylpyridinium and cucurbit [6] uril. *Chem. Sci.* **10,** 7773-7778 (2019).

34. Gao, R., & Yan, D. Layered host–guest long-afterglow ultrathin nanosheets: high-efficiency phosphorescence energy transfer at 2D confined interface. *Chem. Sci.* **8,** 590-599 (2017).

35. Yang, X., & Yan, D. Long-afterglow metal–organic frameworks: reversible

guest-induced phosphorescence tunability. *Chem. Sci.* **7,** 4519-4526 (2016).
